# Insulin-like growth factor 2 mRNA-binding protein 3 promotes kidney injury by regulating **β**-catenin signaling

**DOI:** 10.1172/jci.insight.162060

**Published:** 2023-01-24

**Authors:** Dongyan Song, Jingyue Shang, Yinyi Long, Menghua Zhong, Li Li, Jiongcheng Chen, Yadie Xiang, Huishi Tan, Haili Zhu, Xue Hong, Fan Fan Hou, Haiyan Fu, Youhua Liu

**Affiliations:** State Key Laboratory of Organ Failure Research, National Clinical Research Center of Kidney Disease, Guangdong Provincial Institute of Nephrology, and Division of Nephrology, Nanfang Hospital, Southern Medical University, Guangzhou, China.

**Keywords:** Nephrology, Chronic kidney disease

## Abstract

Wnt/β-catenin is a developmental signaling pathway that plays a crucial role in driving kidney fibrosis after injury. Activation of β-catenin is presumed to be regulated through the posttranslational protein modification. Little is known about whether β-catenin is also subjected to regulation at the posttranscriptional mRNA level. Here, we report that insulin-like growth factor 2 mRNA-binding protein 3 (IGF2BP3) plays a pivotal role in regulating β-catenin. IGF2BP3 was upregulated in renal tubular epithelium of various animal models and patients with chronic kidney disease. IGF2BP3 not only was a direct downstream target of Wnt/β-catenin but also was obligatory for transducing Wnt signal. In vitro, overexpression of IGF2BP3 in kidney tubular cells induced fibrotic responses, whereas knockdown of endogenous IGF2BP3 prevented the expression of injury and fibrosis markers in tubular cells after Wnt3a stimulation. In vivo, exogenous IGF2BP3 promoted β-catenin activation and aggravated kidney fibrosis, while knockdown of IGF2BP3 ameliorated renal fibrotic lesions after obstructive injury. RNA immunoprecipitation and mRNA stability assays revealed that IGF2BP3 directly bound to β-catenin mRNA and stabilized it against degradation. Furthermore, knockdown of IGF2BP3 in tubular cells accelerated β-catenin mRNA degradation in vitro. These studies demonstrate that IGF2BP3 promotes β-catenin signaling and drives kidney fibrosis, which may be mediated through stabilizing β-catenin mRNA. Our findings uncover a previously underappreciated dimension of the complex regulation of Wnt/β-catenin signaling and suggest a potential target for therapeutic intervention of fibrotic kidney diseases.

## Graphical abstract

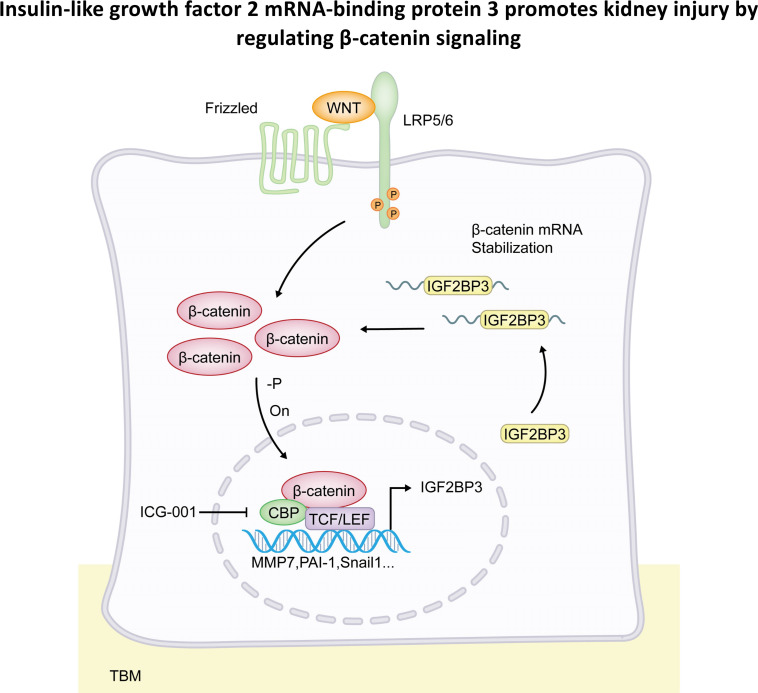

## Introduction

Chronic kidney disease (CKD), characterized by progressive tissue fibrosis and gradual loss of kidney function, is becoming a major public health problem worldwide (1). CKD is highly prevalent and associated with increased risk of progression to end-stage renal disease, a devastating condition with high morbidity and mortality (2). Extensive studies show that kidney fibrosis is the common outcome of all kinds of CKD, regardless of the initial causes (3). The pathophysiology of kidney fibrosis is complex and involves many types of cells, in which the role of tubular epithelial cells is particularly intriguing, because they are the primary targets of kidney injury in most circumstances (4–6). In response to various insults, tubular cells undergo different changes, such as partial epithelial-mesenchymal transition, cell cycle arrest, cellular senescence, or metabolic reprogramming (7–13). These responses are controlled by several key signal cascades, including Wnt/β-catenin signaling.

Wnt/β-catenin is an evolutionarily conserved signaling pathway that is activated after tissue injury and plays a crucial role in driving tissue fibrosis (14). As a master transcriptional regulator, β-catenin drives kidney fibrogenesis by inducing a variety of fibrosis-related downstream targets, such as Snail1, fibronectin, matrix metalloproteinase-7 (MMP-7), plasminogen activator inhibitor-1 (PAI-1), and components of the renin-angiotensin system (15). Consistently, inhibition of β-catenin ameliorates renal fibrotic lesions in various models of CKD (16–18). These findings underscore that hyperactive β-catenin could be the causative culprit behind kidney damage and disease after injury. It is widely accepted that the regulation of β-catenin is primarily controlled at the posttranslational level. In quiescent state, β-catenin is constitutively destructed by a phosphorylation-triggered, ubiquitin-mediated protein degradation. Upon Wnt activation, β-catenin is dephosphorylated, leading to its stabilization, accumulation, and nuclear translocation (19, 20). However, whether β-catenin is also subjected to regulation at the mRNA level is largely unknown. Furthermore, what controls β-catenin mRNA regulation and how important it is in the pathogenesis of CKD remain enigmatic.

Insulin-like growth factor 2 mRNA-binding protein 3 (IGF2BP3) belongs to a highly conserved family of RNA-binding proteins, which also includes IGF2BP1 and IGF2BP2 (21–26). As RNA-binding proteins, IGF2BPs regulate many biological processes, such as embryonic development, tumor formation, and pathogenesis of human diseases, by interacting with various target RNAs, thereby controlling their stability, storage, nuclear export, and subcellular localization and degradation and affecting gene expression output (25, 27, 28). IGF2BPs share a high degree of homology in their sequences, but each of them exhibits uniqueness in terms of expression pattern, target specificity, and injury responses (22, 25). For example, IGF2BP2 has been implicated as a candidate gene involved in type 2 diabetes and has diverse functions in cell metabolism (29, 30), whereas IGF2BP3 is associated with liver fibrosis through controlling transformation of hepatic stellate cells into myofibroblasts (31). IGF2BP3 has been shown to be a prognostic and diagnostic biomarker and therapeutic target for renal cell carcinoma (32–35). However, its role in the pathogenesis of kidney fibrosis remains unknown.

In this study, we show that IGF2BP3 is induced in the kidneys of animal models and patients with CKD. Overexpression of IGF2BP3 impairs tubular cell integrity and promotes kidney fibrosis, possibly by stabilizing β-catenin mRNA. These studies identify IGF2BP3 as a major player in kidney fibrogenesis.

## Results

### Induction of IGF2BP3 in various animal models of CKD.

**Figure 1:**
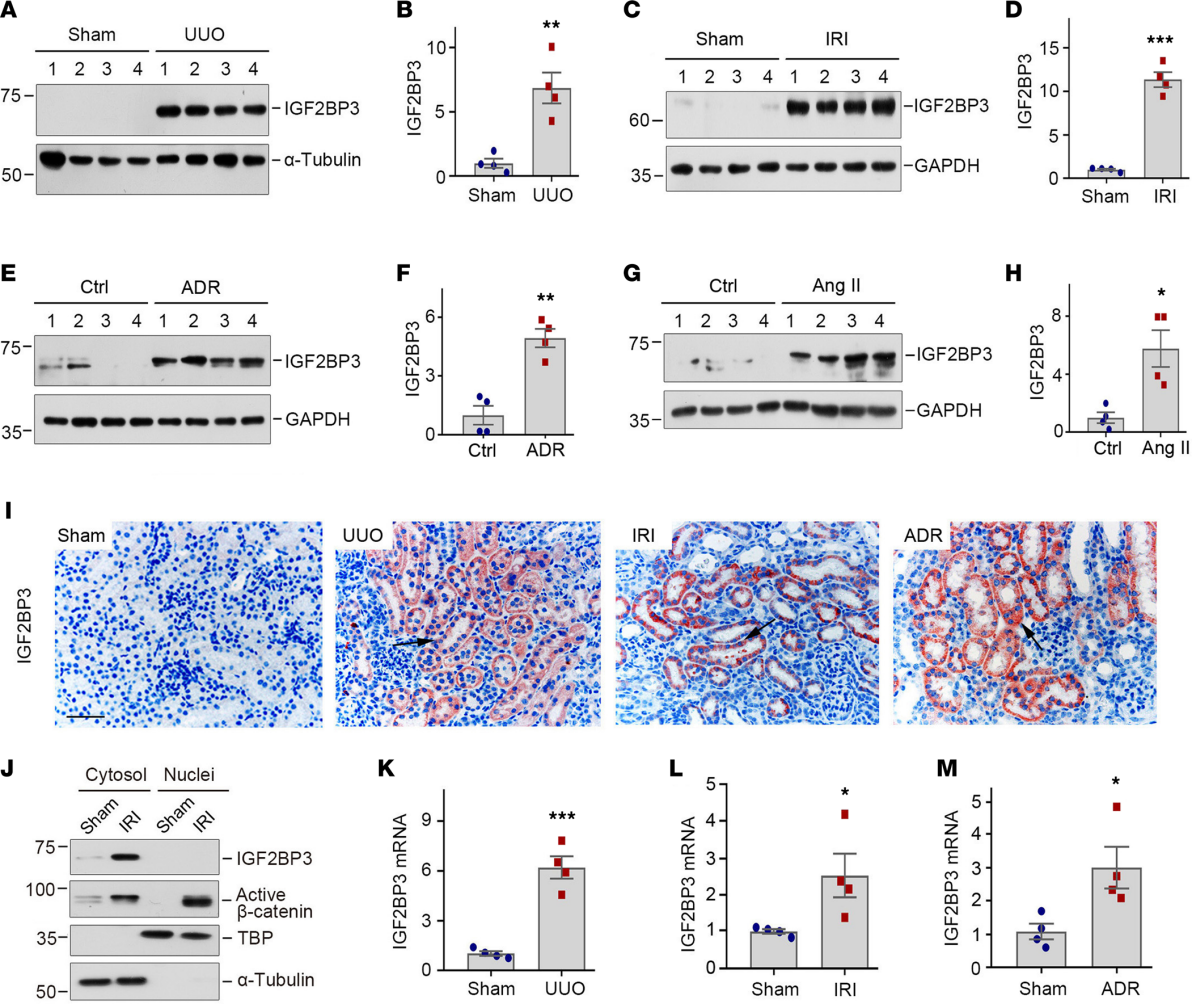
IGF2BP3 is upregulated in renal tubular epithelium in various models of CKD. (**A** and **B**) Western blotting and quantitative determination of IGF2BP3 protein in the kidney at 7 days after UUO. Kidney homogenates after sham and UUO treatment were subjected to Western blotting using anti-IGF2BP3 antibody. **P < 0.01 versus sham (*n* = 4, *t* test). (**C** and **D**) Western blot analyses of renal expression of IGF2BP3 in IRI. IGF2BP3 expression was assessed in the kidneys at 11 days after IRI. ***P < 0.001 versus sham (*n* = 4, *t* test). (**E** and **F**) Western blot analyses of renal expression of IGF2BP3 in ADR. IGF2BP3 expression was assessed in the kidneys at 2 weeks after ADR. **P < 0.01 versus sham (*n* = 4, *t* test). (**G** and **H**) Western blot analyses of renal expression of IGF2BP3 after Ang II infusion. IGF2BP3 expression was assessed in the kidneys at 4 weeks after Ang II infusion. *P < 0.05 versus sham (*n* = 4, *t* test). (**I**) Representative micrographs show renal expression and localization of IGF2BP3 protein in various animal models of CKD. IGF2BP3 was detected by immunohistochemical staining. Arrows indicates positive staining. Scale bar, 50 μm. (**J**) Western blots showed IGF2BP3 and active β-catenin distribution in cytosolic and nuclear fractions at 11 days after IRI. α-Tubulin and TATA-binding protein (TBP) were used to normalize cytosolic and nuclear fractions. (**K**–**M**) qRT-PCR showed renal mRNA levels of IGF2BP3 after UUO (**K**), IRI (**L**), and ADR (**M**), respectively. *P < 0.05, ***P < 0.001 versus sham (*n* = 4, *t* test). Numbers to left of blots indicate kDa.

We first assessed the expression of IGF2BP3 in various well-established animal models of CKD induced by unilateral ureteral obstruction (UUO), ischemia/reperfusion injury (IRI), adriamycin (ADR), and angiotensin II (Ang II), respectively. These models represent diverse etiologies that lead to renal failure and fibrotic lesions. As shown in Figure 1, A–H, IGF2BP3 protein was markedly induced at 7 days after UUO, 11 days after IRI, 2 weeks after ADR, and 4 weeks after chronic Ang II infusion. These results suggest that IGF2BP3 induction is a common pathologic finding in CKD, regardless of the initial causes.

We next examined IGF2BP3 localization in the fibrotic kidneys by using immunohistochemical staining. IGF2BP3 was undetectable in normal kidney but markedly upregulated in CKD (Figure 1I). Strong IGF2BP3 staining was predominantly localized in renal tubular epithelium, whereas glomeruli were essentially negative (Figure 1I). Judging from the staining pattern, IGF2BP3 appeared primarily present in the cytoplasm of tubular epithelial cells. To verify this, we assessed its abundance in the fibrotic kidneys after nuclear and cytoplasmic fractionation. As shown in Figure 1J, IGF2BP3 was upregulated in the cytoplasm, but not in the nuclei, after IRI. In contrast, β-catenin protein was mainly upregulated in the nuclei in the same setting (Figure 1J). Similar induction of renal IGF2BP3 mRNA was evident in the fibrotic kidneys, as demonstrated by quantitative real-time polymerase chain reaction (qRT-PCR) (Figure 1, K–M).

We also assessed the expression of other members of IGF2BP family proteins. IGF2BP2 was also induced in the kidneys after UUO and IRI, although to a lesser extent (Supplemental Figure 1, A, C, D, and F; supplemental material available online with this article; https://doi.org/10.1172/jci.insight.162060DS1). IGF2BP2 localized in renal tubular epithelium as well (Supplemental Figure 1G). The expression of IGF2BP1 was CKD content dependent, and it was induced in the kidney after IRI but not in the fibrotic kidney after UUO (Supplemental Figure 1). Based on these observations, we chose to focus on IGF2BP3 in this study as its induction was most robust and common in all CKD tested.

We found that IGF2BP3 was mainly upregulated in CKD, and little or no induction of IGF2BP3 was observed in animal models of acute kidney injury (AKI) at 3 days after cisplatin or 1 day after IRI (Supplemental Figure 2), suggesting that it could be relevant to kidney fibrogenesis but not to renal repair and regeneration.

### IGF2BP3 is upregulated and associated with kidney dysfunction and fibrosis in human CKD.

**Figure 2:**
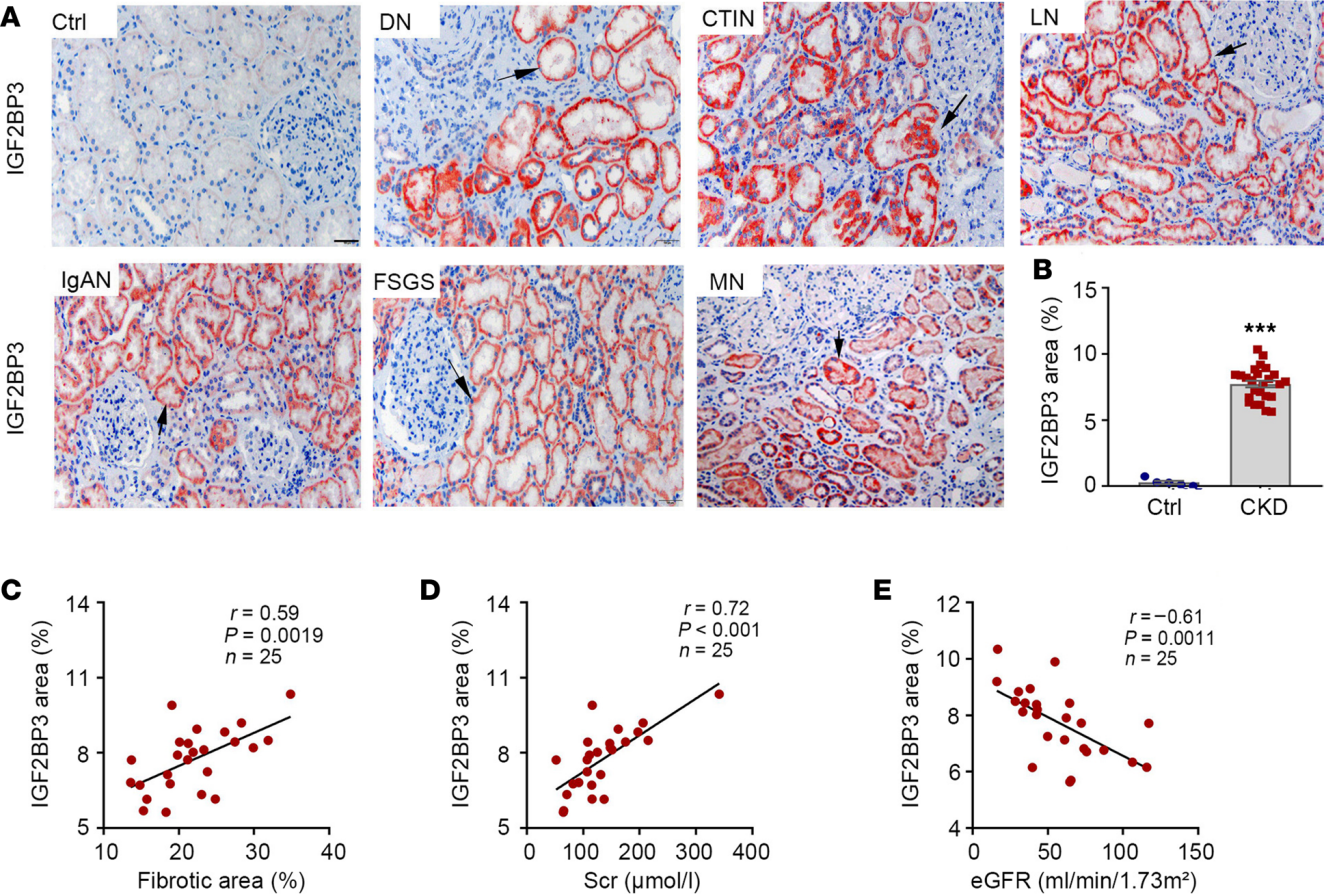
IGF2BP3 is upregulated and associated with kidney dysfunction and fibrosis in human CKD. (**A**) Representative micrographs show the expression and localization of IGF2BP3 protein in human CKD biopsy specimens as indicated. Arrows indicate positive staining. Scale bar, 50 μm. Ctrl, nontumor kidney section from patients with renal cell carcinoma; DN, diabetic nephropathy; CTIN, chronic tubulointerstitial nephritis; LN, lupus nephritis; IgAN, immunoglobulin A nephropathy; FSGS, focal segmental glomerulosclerosis; MN, membranous nephropathy. (**B**) Semiquantitative determination of IGF2BP3-positive area in different groups was shown. ***P < 0.001 versus controls (*n* = 5–25, *t* test). (**C** and **D**) Linear regression shows significant correlation between IGF2BP3-positive area and fibrotic area (**C**) and serum creatinine (Scr) (**D**). (**E**) Linear regression shows a negative correlation between IGF2BP3 area and kidney function (eGFR).

To study the clinical relevance of these findings, we investigated IGF2BP3 expression in human kidney biopsies from patients with CKD. As shown in Figure 2, A and B, IGF2BP3 protein was barely detectable in 5 cases of control, nontumor kidney tissue specimens. However, IGF2BP3 protein was readily observed in all 25 biopsy specimens from patients with different CKDs, albeit with varying staining intensities. The demographic and clinical data of the patients with CKD are presented as Supplemental Table 1. It appeared that IGF2BP3 was predominantly induced in renal tubular epithelium, whereas its glomerular staining was not seen or was minimal (Figure 2A). Representative micrographs of IGF2BP3 staining in kidney biopsy specimens from patients with DN, CTIN, LN, IgAN, FSGS, and MN are presented in Figure 2A. The relative levels of IGF2BP3 protein in control and CKD groups are shown in Figure 2B.

We further assessed fibrotic lesions in the kidney biopsies of patients with CKD after Masson’s trichrome staining and analyzed the relationship between IGF2BP3 and fibrotic lesions and kidney function in 25 cases of CKD. As shown in Figure 2C, IGF2BP3 protein was correlated with the severity of fibrotic lesions in these patients. Similarly, IGF2BP3 was also closely correlated with serum creatinine levels (Figure 2D). Consistently, IGF2BP3 was inversely associated with estimated glomerular filtration rate (eGFR) (Figure 2E).

### IGF2BP3 is a downstream target of Wnt/β-catenin signaling in vitro and in vivo.

**Figure 3:**
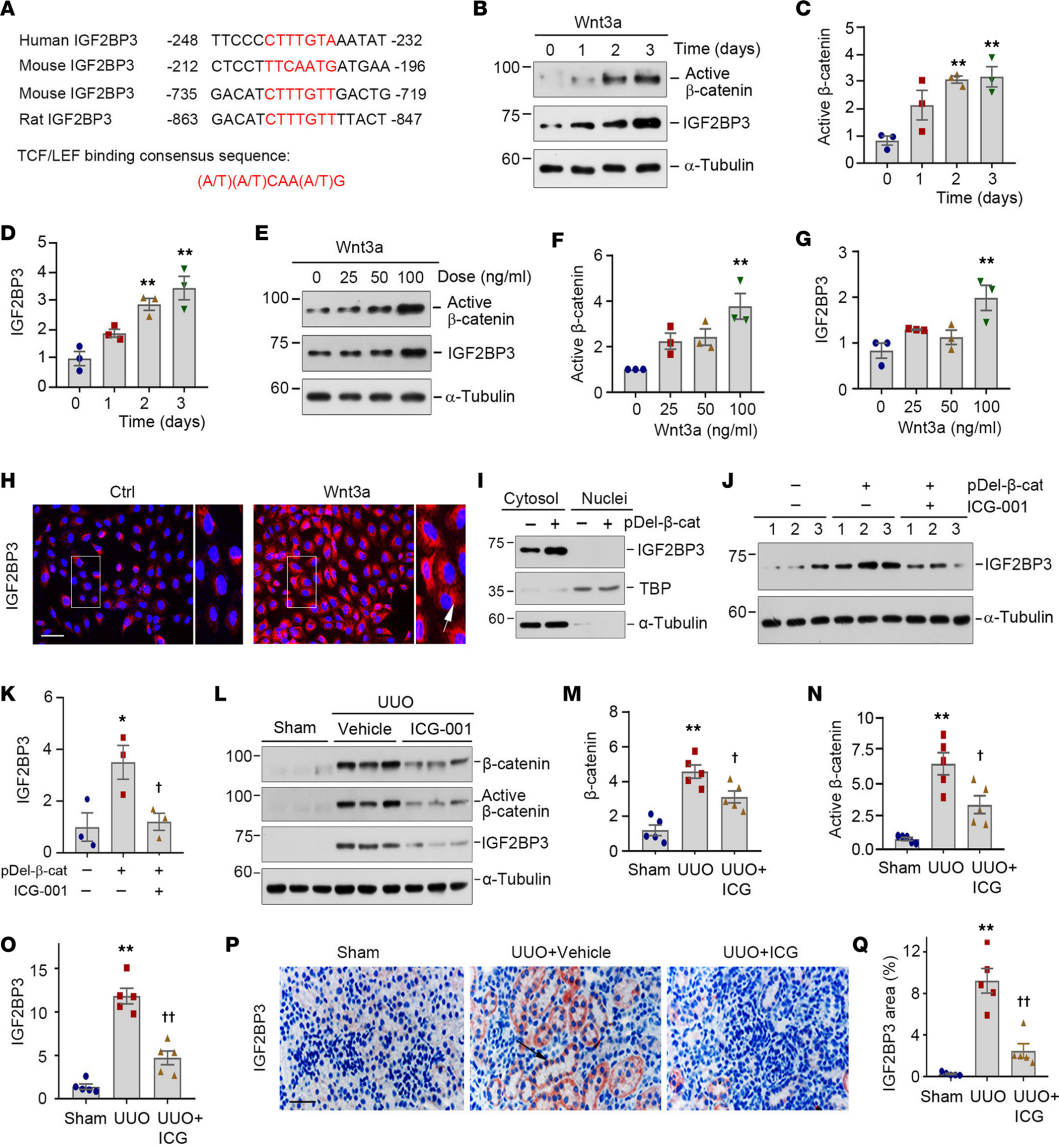
IGF2BP3 is a downstream target of Wnt/β-catenin signaling. (**A**) Bioinformatics analyses revealed the presence of putative TBSs in the promoter region of human, mouse, and rat *IGF2BP3* gene. The sequences and positions of the putative TBSs were highlighted (red), whereas the TBS consensus sequence was given at the bottom of this panel. TBS, TCF/lymphoid enhancer factor binding site. (**B**–**D**) Wnt3a (100 ng/mL) induced active β-catenin and IGF2BP3 expression in HKC-8 cells in a time-dependent manner. Representative Western blot (**B**) and quantitative data (**C** and **D**). **P < 0.01 versus controls (*n* = 3, ANOVA with Student-Newman-Keuls test). (**E**–**G**) Wnt3a induced active β-catenin and IGF2BP3 expression in a dose-dependent fashion. Representative Western blot (**E**) and quantitative data (**F** and **G**). **P < 0.01 versus controls (*n* = 3, ANOVA with Student-Newman-Keuls test). (**H**) Immunofluorescence staining for IGF2BP3 expression after Wnt3a stimulation. Cells were stained for IGF2BP3 at 2 days after incubation with Wnt3a. Boxed areas were enlarged. Arrows indicate positive staining. Scale bar, 50 μm. (**I**) Western blots show IGF2BP3 distribution in cytosolic and nuclear fractions. α-Tubulin and TBP were probed to normalize cytosolic and nuclear fractions. (**J** and **K**) Representative Western blot (**J**) and quantitative data (**K**) show the expression of IGF2BP3 after various treatments. *P < 0.05 versus controls; ^†^*P* < 0.05 versus pDel-β-cat group alone (*n* = 3, ANOVA with Student-Newman-Keuls test). (**L**–**O**) ICG-001 reduced renal expression of β-catenin, active β-catenin, and IGF2BP3 protein at 7 days after UUO. Representative Western blots (**L**) and quantitative data (**M**–**O**). **P < 0.01 versus sham; ^†^*P* < 0.05, ^††^*P* < 0.01 versus the UUO injected with vehicle group (*n* = 5, ANOVA with Student-Newman-Keuls test). (**P**) Representative micrographs show IGF2BP3 protein in different groups as indicated. Arrows indicate positive staining. Scale bar, 50 μm. (**Q**) Quantitative determination of IGF2BP3 staining in different groups. **P < 0.01 versus sham; ^††^*P* < 0.01 versus the UUO injected with vehicle group (*n* = 5, ANOVA with Student-Newman-Keuls test).

We next investigated the potential mechanism underlying IGF2BP3 induction in diseased kidneys. Because activation of Wnt/β-catenin is a common finding in virtually all CKD, this prompted us to examine its role in regulating IGF2BP3 expression. We first analyzed the regulatory regions of the *IGF2BP3* promoter by a bioinformatics approach. As shown in Figure 3A, there were putative TBSs in the promoter region of human, mouse, and rat *IGF2BP3* genes, which were perfectly matched with TBS consensus sequences (Figure 3A).

To ascertain the regulation of *IGF2BP3* by Wnt/β-catenin, HKC-8 cells were incubated with Wnt3a for various periods. As shown in Figure 3, B–D, Wnt3a induced active β-catenin and IGF2BP3 expression in a time-dependent manner. Similarly, Wnt3a also dose-dependently induced active β-catenin and IGF2BP3 expression (Figure 3, E–G). The subcellular localization of IGF2BP3 was assessed by immunostaining. IGF2BP3 predominantly localized in the cytoplasm of HKC-8 cells (Figure 3H). This result was further verified by immunoblotting of cytosolic and nuclear fractionations (Figure 3I). After transfection with N-terminus–truncated, constitutively activated β-catenin expression vector (pDel-β-cat), a substantial upregulation of IGF2BP3 was observed only in the cytosolic fraction (Figure 3I).

To further validate the involvement of Wnt/β-catenin in regulating IGF2BP3, we utilized a specific small molecule (ICG-001) that selectively inhibited β-catenin–mediated gene transcription in HKC-8 cells. As shown in Figure 3, J and K, ICG-001 abolished IGF2BP3 expression induced by activated β-catenin. Similarly, in a mouse model of UUO, ICG-001 also largely abolished β-catenin activation and IGF2BP3 induction in vivo, as shown by Western blotting and immunostaining (Figure 3, L–Q).

### Overexpression of IGF2BP3 activates β-catenin signaling in vitro.

**Figure 4:**
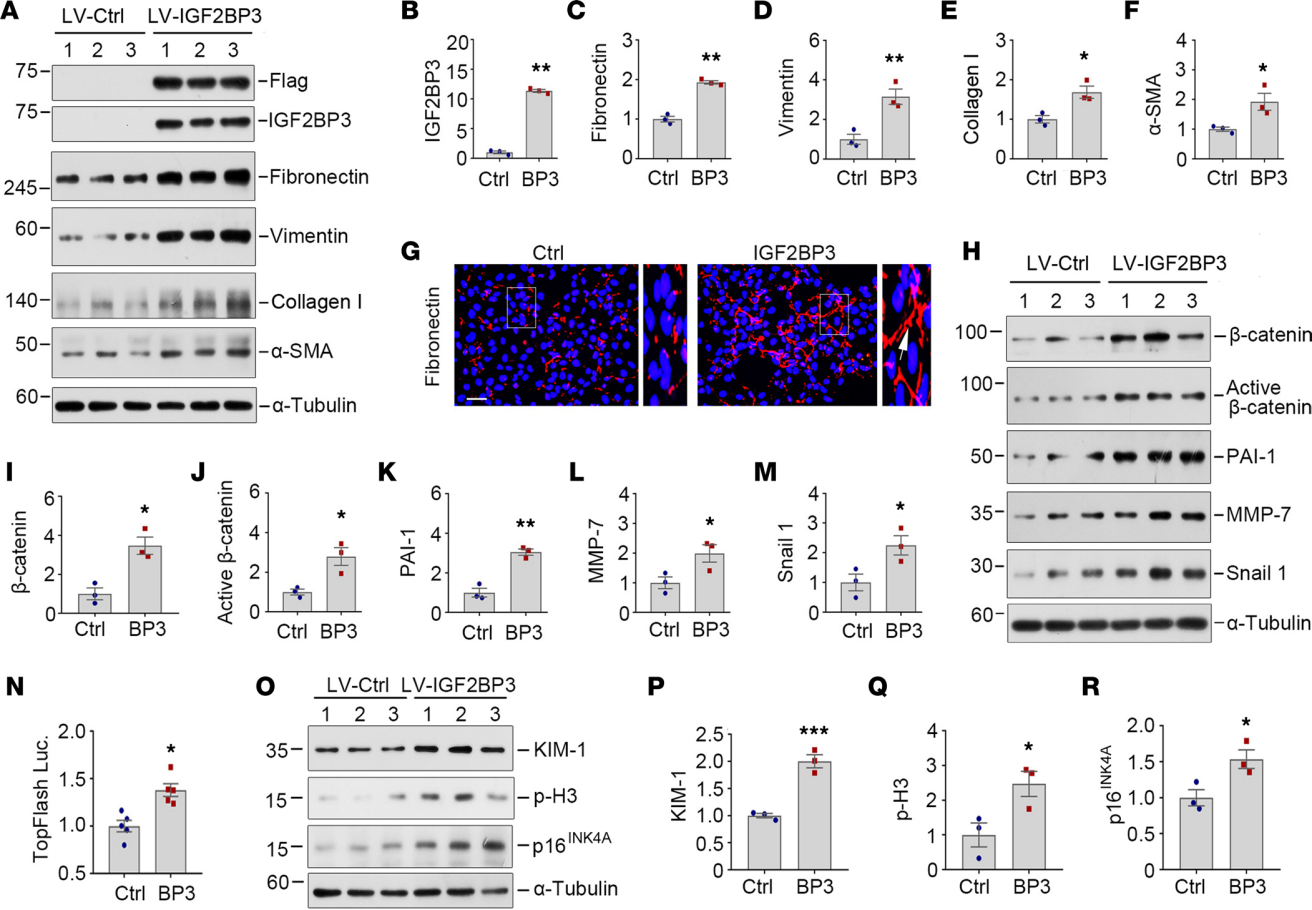
IGF2BP3 induces profibrotic responses and activates β-catenin signaling in vitro. (**A**–**F**) Overexpression of IGF2BP3 triggers the upregulation of fibronectin, vimentin, collagen I, and α-SMA expression in tubular cells. HKC-8 cells were infected with either control lentivirus or IGF2BP3 lentivirus overnight, then incubated for 2 days as indicated. Representative Western blots (**A**) and quantitative data of IGF2BP3 (**B**), fibronectin (**C**), vimentin (**D**), collagen I (**E**), and α-SMA (**F**) are shown. *P < 0.05, **P < 0.01 versus controls (*n* = 3, *t* test). (**G**) Immunofluorescence staining shows fibronectin induction by IGF2BP3 in HKC-8 cells. Cells were stained for fibronectin at 2 days after infection with control lentivirus or IGF2BP3 lentivirus. Boxed areas were enlarged. Arrows indicated positive staining. Scale bar, 50 μm. (**H**–**M**) IGF2BP3 activates β-catenin and active β-catenin and induces its downstream target genes. HKC-8 cells were infected with either control lentivirus or IGF2BP3 lentivirus overnight, then incubated for 2 days as indicated. Representative Western blots (**H**) and quantitative determination of β-catenin (**I**), active β-catenin (**J**), PAI-1 (**K**), MMP-7 (**L**), and Snail1 (**M**) in HKC-8 cells. *P < 0.05, **P < 0.01 versus controls (*n* = 3, *t* test). (**N**) IGF2BP3 activates β-catenin–mediated gene transcription. The HEK293T cells were infected with either control lentivirus or IGF2BP3 lentivirus, followed by transfection with the TOPFlash reporter plasmid. TOPFlash reporter luciferase activities were measured. The relative luciferase activity (fold-change) was reported. *P < 0.05 versus controls (*n* = 5, *t* test). (**O**–**R**) Overexpression of IGF2BP3 triggers the upregulation of KIM-1, p-H3, and p16^INK4A^ expression in tubular cells. HKC-8 cells were infected with either control lentivirus or IGF2BP3 lentivirus overnight, then incubated for 2 days as indicated. Representative Western blots (**O**) and quantitative data of KIM-1 (**P**), p-H3 (**Q**), and p16^INK4A^ (**R**) are shown. *P < 0.05, ***P < 0.001 versus controls (*n* = 3, *t* test).

To delineate the role of IGF2BP3 in tubular cell biology, we examined the effect of its overexpression on kidney tubular epithelial cells. To this end, HKC-8 cells were infected with either control lentivirus (LV-Ctrl) or IGF2BP3-expressing lentivirus (LV-IGF2BP3) overnight and then incubated for 2 days as indicated. As shown in Figure 4, A and B, infection with LV-IGF2BP3 resulted in marked induction of IGF2BP3 or Flag-tag. Interestingly, compared with the LV-Ctrl, overexpression of IGF2BP3 induced the expression of fibronectin, vimentin, collagen I, and α–smooth muscle actin (α-SMA) (Figure 4, A and C–F). Immunostaining for fibronectin gave rise to similar results (Figure 4G).

We found that overexpression of IGF2BP3 induced β-catenin and active β-catenin (Figure 4, H–J), suggesting its role in activating β-catenin. As shown in Figure 4, H and K–M, several downstream targets of β-catenin, such as PAI-1, MMP-7, and Snail1, were also upregulated after overexpression of IGF2BP3. Furthermore, IGF2BP3 activated β-catenin–mediated gene transcription in the TOPFlash reporter luciferase assay in HEK293T cells (Figure 4N). However, we found that IGF2BP3 did not affect TGF-β signaling, as overexpression of IGF2BP3 showed little effect on the expression of TGF-β1, active TGF-β1, TGF-β receptor I, TGF-β receptor II, and total and active Smad2 and Smad3 (Supplemental Figure 3). Therefore, these results indicate that overexpression of IGF2BP3 induces profibrotic responses in tubular epithelial cells primarily by activating β-catenin signaling.

We also examined the effect of IGF2BP3 on cell cycle arrest and cellular senescence, as they contribute to the pathogenesis of kidney injury and fibrosis. As shown in Figure 4, O–R, compared with the LV-Ctrl, overexpression of IGF2BP3 induced the expression of kidney injury molecule-1 (KIM-1) (a tubular injury marker), phosphorylated histone H3 (p-H3) (a G2/M arrest–related marker), and p16^INK4A^ (a cellular senescence–related marker). To clarify whether these effects are mediated through IGF2BP3 regulation of β-catenin, HKC-8 cells were infected with either control lentivirus or IGF2BP3 lentivirus overnight, then incubated with ICG-001. As shown in Supplemental Figure 4, ICG-001 completely blocked IGF2BP3-induced upregulation of active β-catenin, fibronectin, vimentin, KIM-1, and p-H3. These data suggest that β-catenin plays a critical role in mediating the effects of IGF2BP3 on tubular injury, cell cycle arrest, and fibrotic response.

### IGF2BP3 is required for Wnt/β-catenin signaling in vitro.

**Figure 5:**
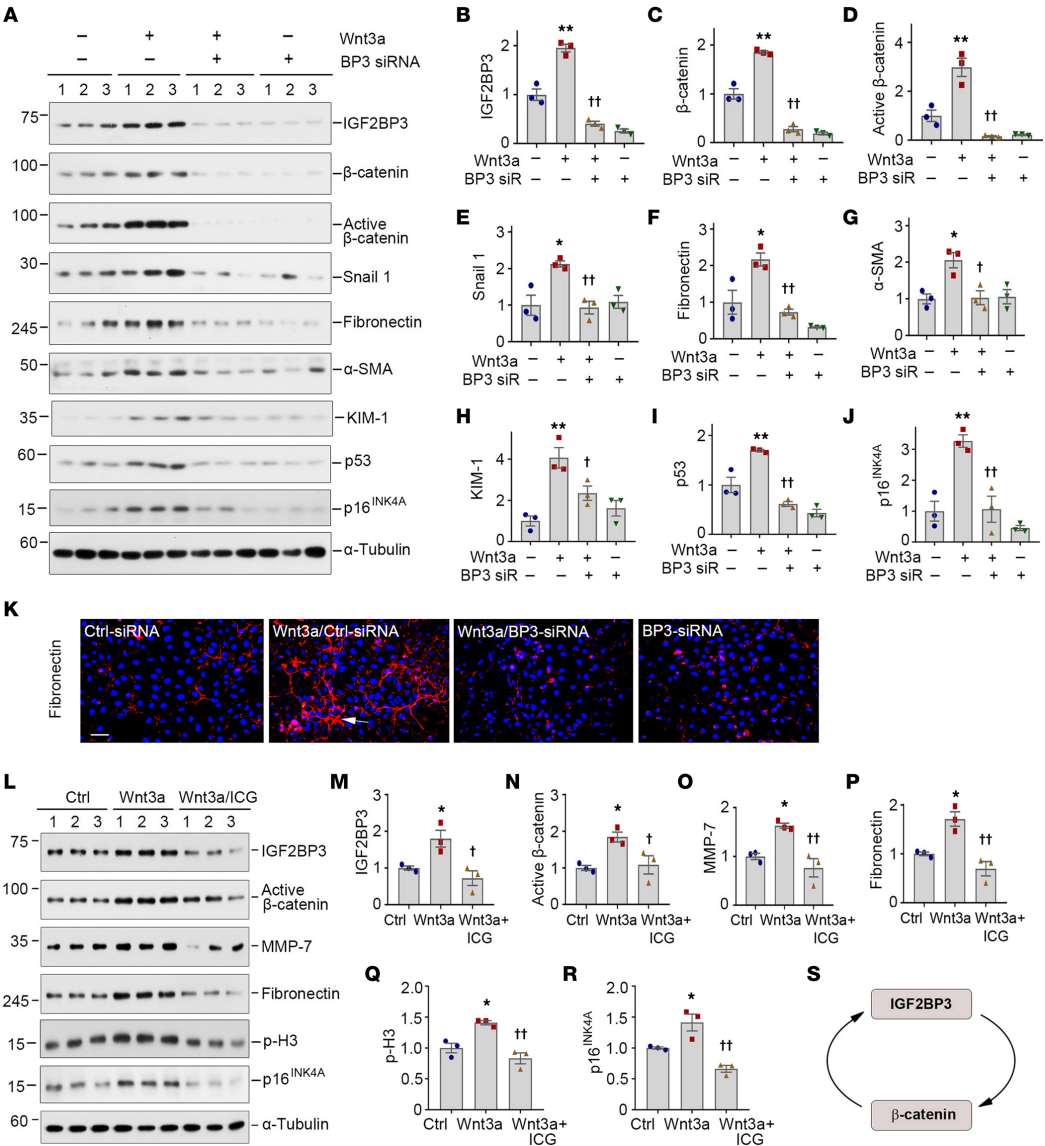
IGF2BP3 is required for mediating Wnt/β-catenin signaling in vitro. (**A**–**J**) Knockdown of IGF2BP3 inhibited Wnt/β-catenin signaling. HKC-8 cells were incubated with Wnt3a (100 ng/mL) in the presence of control siRNA or IGF2BP3-specific siRNA for 2 days, as indicated. Representative Western blot (**A**) and quantitative data for IGF2BP3 (**B**), β-catenin (**C**), active β-catenin (**D**), Snail1 (**E**), fibronectin (**F**), α-SMA (**G**), KIM-1 (**H**), p53 (**I**), and p16^INK4A^ (**J**) are presented. *P < 0.05, **P < 0.01 versus controls; ^†^*P* < 0.05, ^††^*P* < 0.01 versus the Wnt3a plus control siRNA group (*n* = 3, ANOVA with Student-Newman-Keuls test). (**K**) Immunofluorescence staining shows fibronectin expression after various treatments. HKC-8 cells were incubated with Wnt3a (100 ng/mL) in the presence of control siRNA or BP3 siRNA for 2 days as indicated. Arrows indicate positive staining. Scale bar, 50 μm. (**L**–**R**) Representative Western blots (**L**) and quantitative data show the expression of IGF2BP3 (**M**), active β-catenin (**N**), MMP-7 (**O**), fibronectin (**P**), p-H3 (**Q**), and p16^INK4A^ (**R**). HKC-8 cells were pretreated with ICG-001 (10 μM) for 1 hour, then incubated with Wnt3a (100 ng/mL) for 2 days. *P < 0.05 versus controls; ^†^*P* < 0.05, ^††^*P* < 0.01 versus the Wnt3a group (*n* = 3, ANOVA with Student-Newman-Keuls test). (**S**) Schematic presentation of reciprocal feed-forward activation loop between IGF2BP3 and β-catenin.

We wondered whether IGF2BP3 induction is required for Wnt/β-catenin to elicit its profibrotic action. To test this, we transfected HKC-8 cells with control or IGF2BP3-specific siRNA in the absence or presence of Wnt3a. As shown in Figure 5, A and B, transfection of IGF2BP3-specific siRNA knocked down IGF2BP3 induced by Wnt3a in HKC-8 cells. Consistently, knockdown of IGF2BP3 abrogated the Wnt3a-triggered β-catenin activation and its downstream Snail1, fibronectin, α-SMA, KIM-1, p53, and p16^INK4A^ (Figure 5, A and C–J). Immunostaining also showed that depletion of IGF2BP3 abolished Wnt3a-induced fibronectin deposition (Figure 5K). Therefore, IGF2BP3 is required for Wnt3a/β-catenin activation and its downstream genes’ expression.

On the flip side, we found that β-catenin was required for IGF2BP3 induction and function, as ICG-001 abolished the induction of IGF2BP3, activation of β-catenin, and upregulation of MMP-7, fibronectin, p-H3, and p16^INK4A^ after Wnt3a stimulation (Figure 5, L–R). Together, IGF2BP3 induction and β-catenin activation form a reciprocal feed-forward loop (Figure 5S).

### Overexpression of IGF2BP3 aggravates kidney fibrosis and promotes β-catenin signaling in vivo.

To explore the role of IGF2BP3 in vivo, normal mice were injected with control adeno-associated viral vector (AAV-Ctrl) or IGF2BP3 adeno-associated viral vector (AAV-BP3) into 6 sites of the left renal cortex for 12 weeks, as intraparenchymal AAV injections result in robust but relatively local transduction (36). Immunostaining and Western blotting of whole-kidney homogenates revealed that injections of AAV-BP3 vectors induced renal tubular expression of Flag-tagged IGF2BP3 (Supplemental Figure 5). However, overexpression of IGF2BP3 by intrarenal injections of AAV-BP3 did not trigger fibrotic response in normal mice (Supplemental Figure 5).

**Figure 6:**
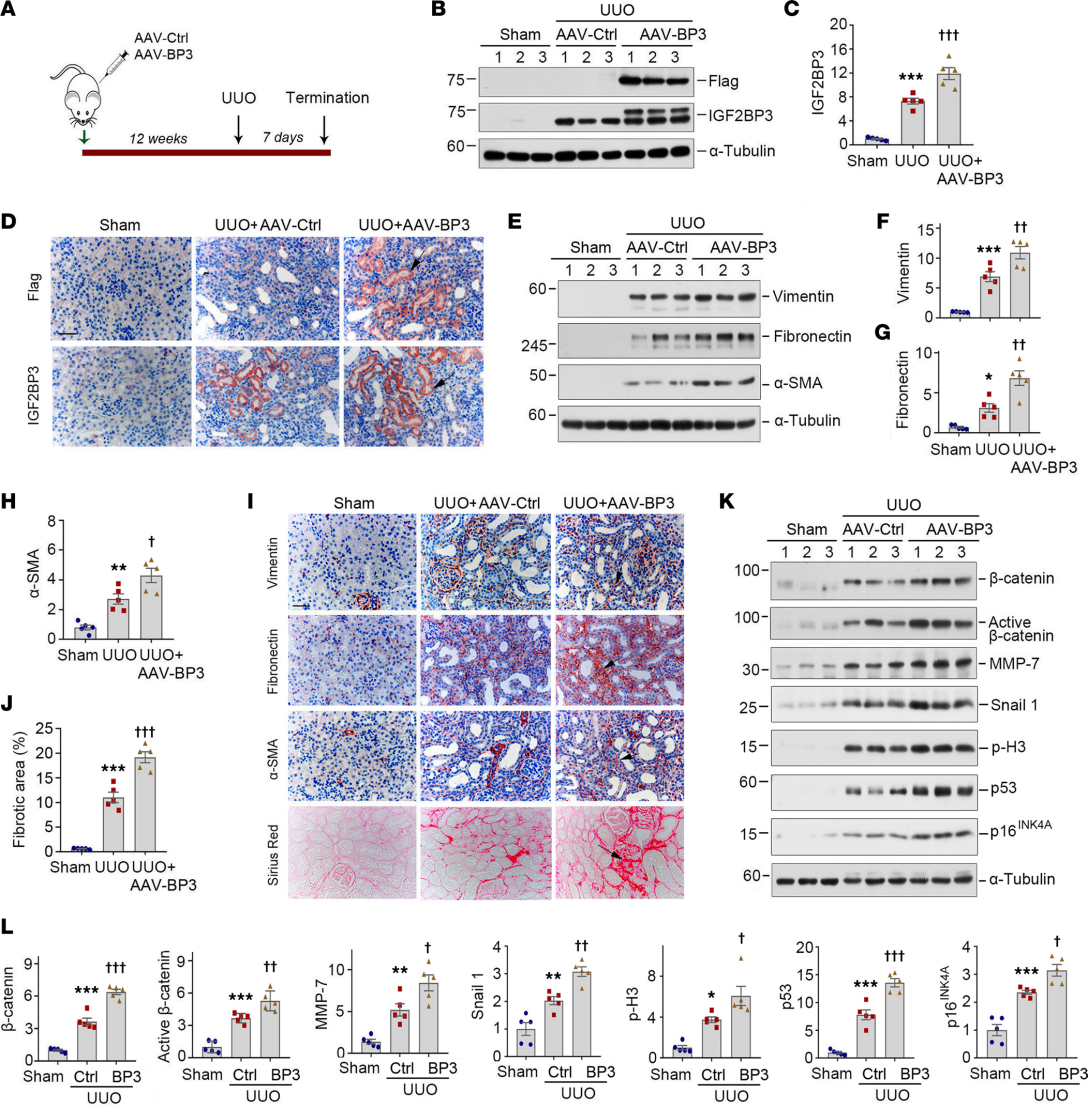
Overexpression of IGF2BP3 in vivo promotes kidney fibrosis and β-catenin activation after UUO. (**A**) Experimental design. The green arrow indicates the timing of injecting AAV-Ctrl or AAV-BP3. The black arrow indicates the timing of UUO surgery. (**B** and **C**) Representative Western blot (**B**) and quantitative data (**C**) show the expression of Flag and IGF2BP3 in different groups as indicated. ***P < 0.001 versus the sham injected with AAV-Ctrl group; ^†††^*P* < 0.001 versus the UUO injected with AAV-Ctrl group (*n* = 5, ANOVA with Student-Newman-Keuls test). (**D**) Micrographs show Flag and IGF2BP3 expression in UUO kidney. Arrows indicate positive staining. Scale bar, 50 μm. (**E**–**H**) Representative Western blot (**E**) and quantitative data show the expression of vimentin (**F**), fibronectin (**G**), and α-SMA (**H**) proteins in different groups as indicated. *P < 0.05, **P < 0.01, ***P < 0.001 versus the sham-injected with AAV-Ctrl group; ^†^*P* < 0.05, ^††^*P* < 0.01 versus the UUO injected with AAV-Ctrl group (*n* = 5, ANOVA with Student-Newman-Keuls test). (**I**) Micrographs show the expression of vimentin, fibronectin, α-SMA, and collagens at 7 days after UUO in different groups. Arrows indicate positive staining. Scale bar, 50 μm. (**J**) Graphical presentation of renal fibrotic lesions in different groups. ***P < 0.001 versus the sham injected with AAV-Ctrl group; ^†††^*P* < 0.001 versus the UUO injected with AAV-Ctrl group (*n* = 5, ANOVA with Student-Newman-Keuls test). (**K** and **L**) Representative Western blots (**K**) and quantitative data (**L**) show the expression of β-catenin, active β-catenin, MMP-7, Snail1, p-H3, p53, and p16^INK4A^ in different groups as indicated. *P < 0.05, **P < 0.01, ***P < 0.001 versus the sham injected with AAV-Ctrl group; ^†^*P* < 0.05, ^††^*P* < 0.01, ^†††^*P* < 0.001 versus the UUO injected with AAV-Ctrl group (*n* = 5, ANOVA with Student-Newman-Keuls test).

To further investigate the role of IGF2BP3 in the pathogenesis of CKD, we used a mouse model of UUO, a widely used model with robust kidney fibrosis. To this end, mice were injected with AAV-Ctrl or AAV-BP3 at 12 weeks prior to UUO (Figure 6A). Western blotting of whole-kidney homogenates revealed that injections of AAV-BP3 vectors induced renal expression of Flag-tagged IGF2BP3 (Figure 6, B and C). This result was verified by immunostaining for Flag and IGF2BP3, respectively (Figure 6D and Supplemental Figure 6, A and B). Both IGF2BP3 and Flag staining were predominantly localized in cortical tubular epithelium of mouse kidneys (Figure 6D).

We found that overexpression of IGF2BP3 promoted the expression of several fibrosis-related proteins and aggravated kidney fibrotic lesions. As shown in Figure 6, E–H, exogenous IGF2BP3 could upregulate renal vimentin, fibronectin, and α-SMA expression at 7 days after UUO. This result was further verified by immunostaining (Figure 6I and Supplemental Figure 6, C–E). Furthermore, overexpression of IGF2BP3 promoted collagen deposition in UUO kidneys, as shown by Sirius red staining (Figure 6, I and J).

We also investigated the effect of IGF2BP3 on β-catenin signaling in vivo. As shown in Figure 6, K and L, renal expression of β-catenin, active β-catenin, MMP-7, Snail1, p-H3, p53, and p16^INK4A^ was upregulated in UUO mice, and overexpression of IGF2BP3 augmented these inductions.

### Knockdown of IGF2BP3 ameliorates kidney fibrosis and inhibits β-catenin signaling in vivo.

**Figure 7:**
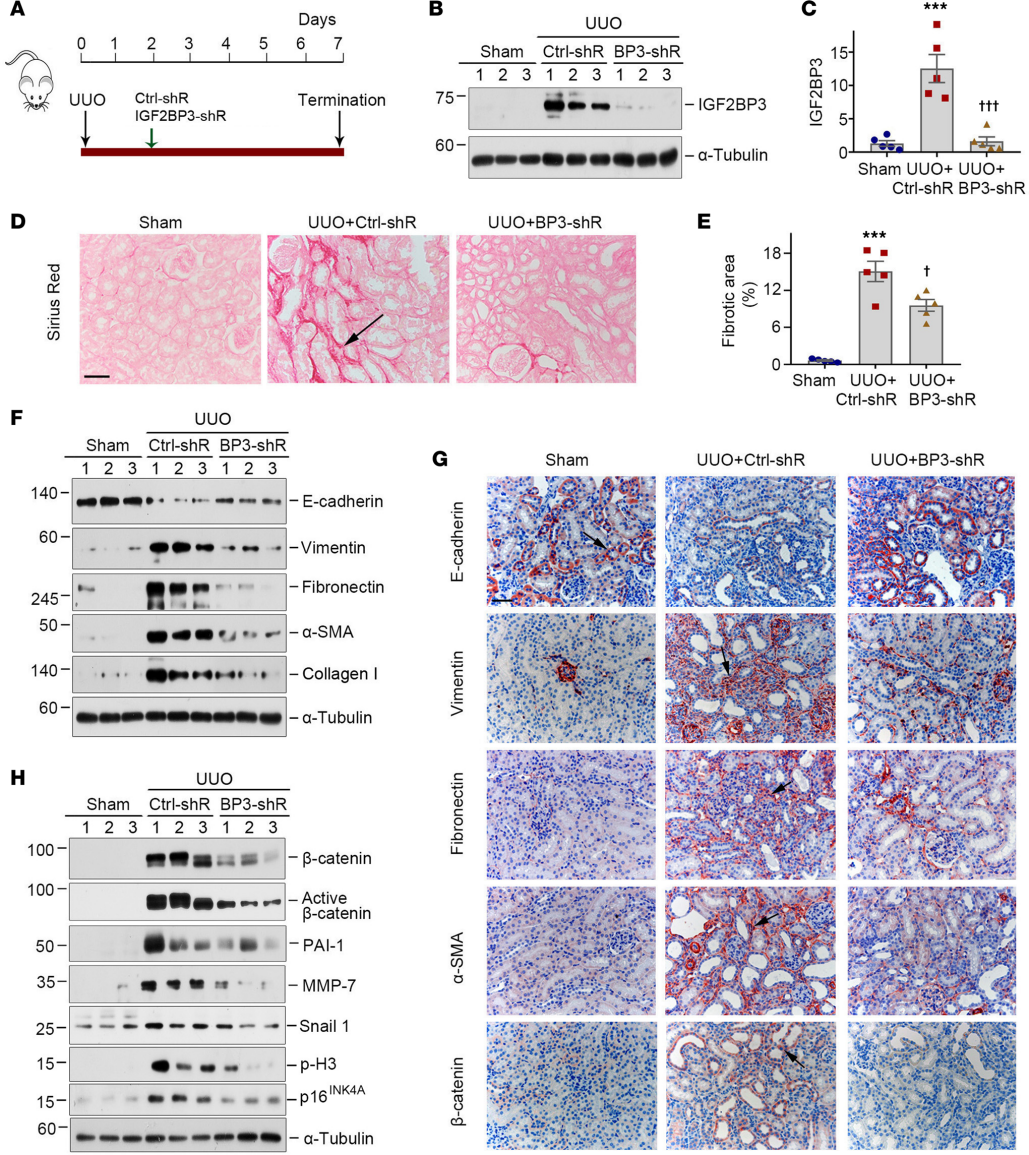
Knockdown of IGF2BP3 ameliorates renal fibrosis and inhibits β-catenin signaling activation after UUO. (**A**) Experimental design. The green arrow indicates the timing of injecting Ctrl-shR or IGF2BP3-shR. The black arrow indicates the timing of UUO. (**B** and **C**) Representative Western blot (**B**) and quantitative data (**C**) show the expression of IGF2BP3 protein in different groups as indicated. ***P < 0.001 versus the sham group; ^†††^*P* < 0.001 versus the UUO injected with Ctrl-shR group (*n* = 5, ANOVA with Student-Newman-Keuls test). (**D**) Micrographs show collagen deposition at 7 days after UUO in different groups. Arrows indicate positive staining. Scale bar, 50 μm. (**E**) Graphical presentation of renal fibrotic lesions in different groups. ***P < 0.001 versus the sham group; ^†^*P* < 0.05 versus the UUO injected with Ctrl-shR group (*n* = 5, ANOVA with Student-Newman-Keuls test). (**F**) Representative Western blots show the expression of E-cadherin, vimentin, fibronectin, α-SMA, and collagen I protein in different groups as indicated. (**G**) Micrographs show E-cadherin, vimentin, fibronectin, α-SMA, and β-catenin at 7 days after UUO in different groups. Arrows indicate positive staining. Scale bar, 50 μm. (**H**) Representative Western blots show the expression of β-catenin, active β-catenin, PAI-1, MMP-7, Snail1, p-H3, and p16^INK4A^ proteins in different groups as indicated.

To further confirm the role of IGF2BP3 in CKD, we sought to knock down IGF2BP3 by using an shRNA-mediated inhibition approach in vivo. Mice were injected intravenously with either Ctrl-shR or IGF2BP3-shR plasmids at 2 days after UUO (Figure 7A). As illustrated in Figure 7, B and C, renal expression of IGF2BP3 was inhibited after intravenous injection of IGF2BP3-shR.

We next assessed the effects of IGF2BP3 depletion on fibrotic lesions after UUO. As shown in Figure 7, D and E, knockdown of IGF2BP3 reduced collagen deposition, as shown by Sirius red staining. Western blotting showed that knockdown of IGF2BP3 inhibited renal expression of vimentin, fibronectin, α-SMA, and collagen I, and restored, at least partially, renal E-cadherin expression in UUO mice (Figure 7F and Supplemental Figure 7, A–E). Immunostaining for E-cadherin, vimentin, fibronectin, and α-SMA proteins gave rise to similar results (Figure 7G and Supplemental Figure 7, F–I).

We also assessed the effects of IGF2BP3 depletion on β-catenin signaling. As shown in Figure 7H and Supplemental Figure 8, A–G, renal expression of β-catenin, active β-catenin, PAI-1, MMP-7, Snail1, p-H3, and p16^INK4A^ was upregulated in UUO mice, whereas knockdown of IGF2BP3 abolished the induction of these proteins. Immunostaining for β-catenin produced similar results (Figure 7G and Supplemental Figure 7J). Knockdown of IGF2BP3 also reduced renal mRNA levels of fibronectin and β-catenin after UUO (Supplemental Figure 8, H and I). Therefore, depletion of IGF2BP3 ameliorates renal fibrosis and inhibits β-catenin activation after UUO.

### IGF2BP3 binds to and stabilizes β-catenin mRNA.

**Figure 8:**
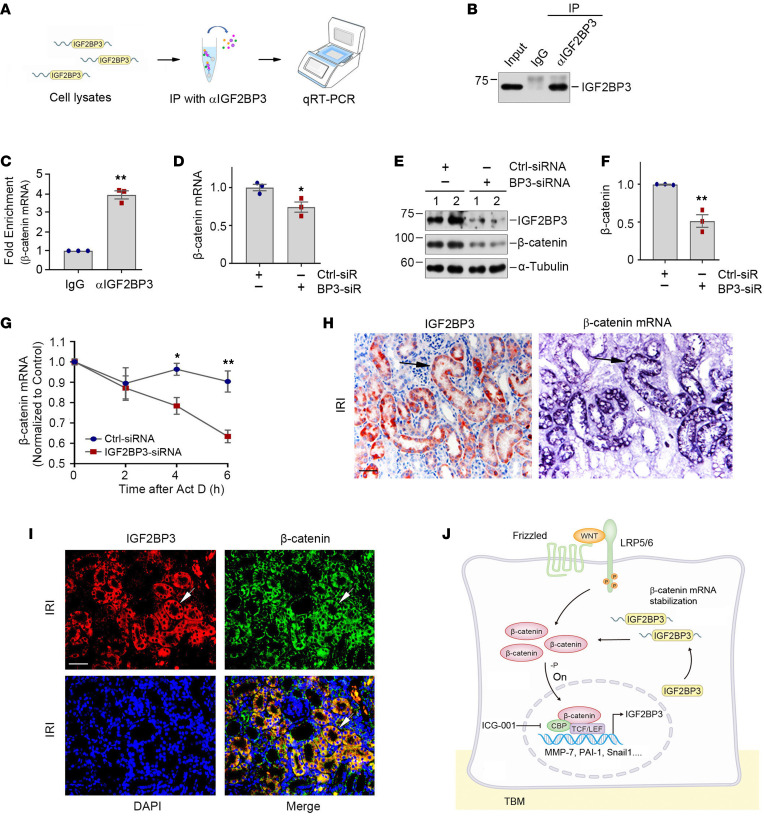
IGF2BP3 binds to β-catenin mRNA and enhances its stability. (**A**) Flowchart shows experimental design and procedures. qRT-PCR was performed with the primers of *CTNNB1* and 18S rRNA. (**B**) Representative Western blots show the protein levels of IGF2BP3 from input, RIP with IgG, or αIGF2BP3. (**C**) Assessment of *CTNNB1* mRNA in RIP by qRT-PCR. 18S rRNA was used for normalization. **P < 0.01 versus IgG control group (*n* = 3, *t* test). (**D**) Quantitative data show that knockdown of IGF2BP3 decreases β-catenin mRNA in HKC-8 cells. β-Actin was used for normalization. *P < 0.05 versus Ctrl siRNA group (*n* = 3, *t* test). (**E** and **F**) Representative Western blots (**E**) and quantitative data (**F**) show the expression of β-catenin protein. **P < 0.01 versus Ctrl siRNA group (*n* = 3, *t* test). (**G**) Knockdown of IGF2BP3 decreases β-catenin mRNA stability. *P < 0.05, **P < 0.01 versus Ctrl siRNA group (*n* = 3, *t* test). (**H**) Colocalization of IGF2BP3 protein and β-catenin mRNA in renal tubules after IRI. Kidney serial sections were stained for IGF2BP3 using immunohistochemical staining and β-catenin mRNA using ISH. Arrows indicate IGF2BP3 and β-catenin mRNA colocalization. Scale bar, 50 μm. (**I**) Colocalization of IGF2BP3 (red) and β-catenin protein (green) in renal tubules after IRI. Arrows indicate IGF2BP3 and β-catenin colocalization. Scale bar, 50 μm. (**J**) Working model of the reciprocal feed-forward activation loop between IGF2BP3 and β-catenin. In tubular epithelial cells, Wnts’ engagement with their receptors leads to β-catenin activation, resulting in induction of IGF2BP3. In turn, upregulated IGF2BP3 protein activates β-catenin signaling by binding to and stabilizing its mRNA. -P, dephosphorylation; CBP, cAMP response element binding protein binding protein; TBM, tubular basement membrane.

We further investigated the potential mechanisms underlying IGF2BP3 regulation of β-catenin. As an RNA-binding protein, IGF2BP3 action might be related to its interaction with target RNAs (21, 22, 28). Accordingly, we tested whether IGF2BP3 directly binds to β-catenin mRNA by performing an RNA immunoprecipitation (RIP) assay. As shown in Figure 8, A–C, β-catenin mRNA was detected in the immunocomplexes precipitated by anti-IGF2BP3 antibody in HKC-8 cells, suggesting a direct interaction between IGF2BP3 and β-catenin mRNA. In addition, knockdown of IGF2BP3 decreased the steady-state levels of β-catenin mRNA and protein in HKC-8 cells, respectively (Figure 8, D–F). Furthermore, knockdown of IGF2BP3 reduced the mRNA stability of β-catenin, as its steady-state level rapidly declined in IGF2BP3-depleted HKC-8 cells when new RNA transcription was inhibited by treatment with actinomycin D (Figure 8G).

We found that IGF2BP3 and β-catenin mRNA and protein were colocalized in the tubular epithelial cells of the fibrotic kidney. As shown in Figure 8H, IGF2BP3 protein was colocalized with β-catenin mRNA by ISH on serial sections. Similarly, IGF2BP3 was colocalized with β-catenin protein by immunostaining (Figure 8I). These results suggest that IGF2BP3 promotes β-catenin expression by binding to and stabilizing its mRNA. As depicted in Figure 8J, IGF2BP3 binds to β-catenin mRNA, leading to its stabilization. This results in increased β-catenin protein expression and its subsequent activation, which in turn induces its downstream target genes, including IGF2BP3.

## Discussion

As the principal mediator of canonical Wnt signaling, β-catenin plays a central role in promoting kidney fibrosis by controlling the expression of a wide variety of fibrosis-related genes (37). The regulation of β-catenin is primarily controlled at the posttranslational level via protein modifications such as phosphorylation and ubiquitination (19). In this study, we show a potentially novel mechanism of β-catenin regulation possibly through stabilization of its mRNA by IGF2BP3, an RNA-binding protein. We show that IGF2BP3 is induced predominantly in kidney tubular epithelium in all CKD models tested, including UUO, IRI, ADR, and Ang II infusion, as well as in human kidney biopsies of patients with various CKDs such as DN, CTIN, LN, IgAN, FSGS, and MN. Overexpression of IGF2BP3 impairs kidney integrity and drives renal fibrosis via activation of β-catenin. Interestingly, IGF2BP3 itself is a direct downstream target of Wnt/β-catenin, but it is also obligatory for Wnt signal transduction. Mechanistically, IGF2BP3 directly binds to β-catenin mRNA and increases its stability. Collectively, IGF2BP3 and β-catenin constitute a reciprocal feed-forward activation loop, which plays a central role in renal fibrogenesis. These studies provide potentially novel insights into the critical role and molecular mechanism of IGF2BP3 in kidney fibrogenesis and underscore the importance of mRNA regulation in β-catenin signaling. These findings expand our understanding of the complex regulation of Wnt/β-catenin and offer a potential new target for therapeutic intervention of fibrotic CKD.

IGF2BPs, like other RNA-binding proteins, are a group of proteins that bind to the single- or double-stranded RNA in cells and participate in forming ribonucleoprotein complexes (25). IGF2BPs contain various structural motifs, such as RNA recognition motif, RNA-binding domain, and K-homology domains (25). They establish highly dynamic interactions with coding and noncoding RNAs and play a specific role in dictating the entire RNA life cycle from alternative splicing to nuclear export, storage, stabilization, subcellular localization, and degradation (25, 27). As such, IGF2BPs are one of the major posttranscriptional regulators of gene expression for fine-tuning protein production. Although the role of IGF2BPs in tumorigenesis is increasingly recognized, the present study represents what we believe is the first comprehensive investigation of IGF2BPs in the pathogenesis of CKD in animal models and in humans. Among these IGF2BP proteins, IGF2BP3 is identified as the one with the most robust and consistent upregulation in diseased kidneys (Figure 1). The induction of IGF2BP1 appears disease specific, which occurs in the kidney after IRI but not UUO, while IGF2BP2 is only moderately induced in both UUO and IRI (Supplemental Figure 1). These observations prompted us to select IGF2BP3 for subsequent investigation in detail. It worthwhile to note that there is a high degree of similarity among these IGF2BPs, suggesting that they could share many biological functions. Consistent with this view, approximately 55%–70% of the recognized target RNAs are shared among 3 IGF2BPs (22). Of interest, IGF2BP3 expression was not changed in the mouse model of AKI induced by cisplatin and only slightly increased after IRI (Supplemental Figure 2), suggesting that it is more relevant to kidney fibrogenesis than renal repair and regeneration after injury.

Regarding the trigger and mechanism governing IGF2BP3 induction in CKD, the present study has illustrated that IGF2BP3 is a downstream target of Wnt/β-catenin (Figure 3). IGF2BP3 is expressed during embryogenesis but largely silent in adult tissues. It is reexpressed in numerous tumors (21, 22). We show here that IGF2BP3 is induced in various animal models and human CKD, which is localized in the cytoplasm of tubular epithelial cells, consistent with previous studies in renal cell carcinoma (32, 34). The expression pattern of IGF2BP3 is reminiscent of β-catenin in diseased kidney, raising the possibility that β-catenin may control its expression. Indeed, there were putative TBSs in the regulatory region of human, mouse, and rat *IGF2BP3* genes. Human Wnt3a or constitutively active β-catenin induced IGF2BP3 expression. Furthermore, inhibition of β-catenin signaling by ICG-001, a small molecule that selectively inhibits β-catenin–mediated gene transcription, blocked IGF2BP3 expression both in vitro and in vivo. The finding that IGF2BP3 is a downstream target of Wnt/β-catenin is also consistent with earlier reports that IGF2BP1 and IGF2BP2 expression is correlated with β-catenin (38–41).

One interesting finding of the present study is that IGF2BP3 elicits its profibrotic actions by activating β-catenin, suggesting that the interplay between IGF2BP3 and β-catenin is bidirectional. In fact, IGF2BP3 is absolutely required for proper Wnt signal transduction in kidney tubular cells, because knockdown of IGF2BP3 abolished β-catenin activation and its target genes’ expression in response to Wnt3a stimulation (Figure 5). As an RNA-binding protein, the action of IGF2BP3 relies on its interaction with its target RNAs (21, 22). Indeed, RIP showed that IGF2BP3 directly binds to and stabilizes β-catenin mRNA in kidney tubular cells. Knockdown of IGF2BP3 decreased the stability and accelerated the degradation of β-catenin mRNA (Figure 8). Furthermore, IGF2BP3 is colocalized with β-catenin mRNA and protein in renal tubular epithelium of the diseased kidney (Figure 8). Taken together, IGF2BP3 and β-catenin create a reciprocal activation loop that synergistically promotes the pathogenesis of renal fibrosis. Our studies also uncover that regulation of mRNA stability could be an important and clinically relevant controlling mechanism of β-catenin signaling in kidney fibrosis. Notably, ectopic expression of IGF2BP3 in normal mice does not cause appreciable kidney injury, suggesting that it is required, but not sufficient, to initiate kidney damage under physiological conditions in vivo.

The present study, however, leaves several questions unanswered. For example, the exact molecular mechanisms by which IGF2BP3 recognizes and regulates β-catenin mRNA remain elusive. Earlier studies have shown that IGF2BPs bind to their target RNAs at the 5′-untranslated region (UTR), the 3′-UTR, or coding regions by recognizing specific RNA motifs (42, 43). The binding sites between IGF2BP3 and β-catenin mRNA in the setting of CKD remain unknown. Furthermore, a recent study has provided compelling evidence showing that IGF2BPs are identified as RNA N^6^-methyladenosine (m^6^A) readers and promote the stability of their mRNA targets in an m^6^A-dependent manner (28). However, whether IGF2BP3-mediated β-catenin mRNA stabilization is also m^6^A-dependent needs to be elucidated. In addition, as IGF2BP3 can potentially bind to many RNA transcripts, the possibility also exists that it may promote kidney fibrosis by interacting with other RNA targets beyond β-catenin (28). In this regard, however, IGF2BP3 does not affect the activity of TGF-β signaling (Supplemental Figure 3). The present study also has several limitations. For example, there is a lack of genetically modified mouse models such as IGF2BP3 knockout or conditional knockout mutants for strengthening the conclusion. Furthermore, only the UUO model was used in the investigation of the impact of different levels of IGF2BP3 on kidney fibrosis in vivo. Clearly, more studies are warranted in the future.

In summary, the present study demonstrates that IGF2BP3, an RNA-binding protein, is upregulated in animal models and patients with CKD and critically involved in kidney fibrogenesis by activating β-catenin signaling. These findings underscore that regulation of β-catenin at the mRNA level, a process that was largely overlooked in the past, is an underappreciated dimension of the complex regulation of Wnt signaling. Although more studies are needed, our studies could provide a potential target for therapeutic intervention of fibrotic CKD.

## Methods

### Animal models.

Male C57BL/6 mice and BALB/c mice, weighing about 20–25 g, were obtained from the Experimental Animal Center of Southern Medical University in Guangzhou, China. For the UUO model, C57BL/6 mice were used as described previously (44). Briefly, UUO was carried out under general anesthesia by ligating the left ureter via 4-0 silk after an abdominal midline incision. At 7 days after UUO, groups of mice were sacrificed, and kidney tissues were collected for subsequent analyses. To investigate renal expression of IGF2BP3 in different AKI and CKD models, mouse models of AKI including IRI and cisplatin injury and CKD including IRI, ADR nephropathy, and chronic Ang II infusion were used. For ischemic AKI mouse models, C57BL/6 mice were subjected to bilateral renal IRI by an established protocol as described previously (45). Briefly, IRI was carried out by clamping bilateral renal pedicles for 30 minutes using microaneurysm clamps. During the ischemic period, body temperature was maintained at 37.5°C by using a temperature-controlled heating system. Mice were sacrificed at 24 hours after IRI and serum and kidney tissues collected for various analyses. For cisplatin injury, C57BL/6 mice were subjected to a single intraperitoneal injection of cisplatin (MilliporeSigma) at a dose of 20 mg/kg as described elsewhere (45). Mice were sacrificed at 3 days after cisplatin injection, and serum and kidney samples were collected for various analyses. For ischemic CKD mouse models, C57BL/6 mice were subjected to unilateral renal IRI by an established protocol as described previously (7). Briefly, IRI was carried out by clamping renal pedicles of the left kidney for 35 minutes using microaneurysm clamps. During the ischemic period, body temperature was maintained at 37.5°C by using a temperature-controlled heating system. At day 10, the contralateral, intact kidney was removed. At day 11 after IRI, groups of mice were sacrificed, and serum and kidney tissues were collected for various analyses. For the ADR model, BALB/c mice were administered with ADR at 10 mg/kg (doxorubicin hydrochloride; MilliporeSigma) by intravenous injection (46). At 2 weeks after ADR injection, mice were euthanized, and kidney tissues were collected for various analyses. For the Ang II infusion model, C57BL/6 mice were implanted with osmotic minipumps (Model 2ML4; Alzet) subcutaneously for chronic administration of Ang II at 0.75 mg/kg/d (47). At 4 weeks after Ang II infusion, mice were euthanized and kidney tissues collected for various analyses.

### Human kidney biopsy samples.

Human kidney biopsy samples were obtained from diagnostic renal biopsies performed at the Nanfang Hospital, Southern Medical University, with written informed consent from the patients. Human normal kidney controls were obtained from nontumor renal tissues of patients who had renal cell carcinoma and underwent nephrectomy. Paraffin-embedded human kidney biopsy sections (3 μm) were prepared using a standard procedure and used for immunohistochemical staining. Quantification was assessed by a computer-aided point-counting technique.

### Cell culture and treatment.

Human kidney proximal tubular cells (HKC-8) were provided by Lorraine C. Racusen (Johns Hopkins University, Baltimore, Maryland, USA). HKC-8 cells were grown in DMEM/Ham’s F12 medium supplemented with 10% fetal bovine serum (FBS). HEK293T cells were obtained from the American Type Culture Collection and cultured in DMEM supplemented with 10% FBS. These cells were cultured at 37°C in an atmosphere containing 5% CO_2_. Serum-starved HKC-8 cells were treated by human recombinant Wnt3a protein (5036-WN-010; R&D Systems, Bio-Techne) at varying dosages in the serum-free medium for various periods as indicated. In some experiments, HKC-8 cells were pretreated with ICG-001 (HY-14428; MedChemExpress) at 10 μM for 1 hours, followed by incubation with vehicle or Wnt3a for an additional 2 days. Cells were then collected and subjected to various analyses.

### Lentivirus infection, plasmid transfection, and siRNA inhibition.

The LV was used to express *IGF2BP3* gene by infecting cells. The recombinant lentivirus vector expressing IGF2BP3, designated as LV-IGF2BP3, was constructed by Hanbio Biotechnology Co. Briefly, the full length of IGF2BP3 was inserted into a pHBLV-CMV-MCS-3xflag-EF1-ZsGreen-T2A-PURO vector. After validation by sequencing, lentivirus was packed, purified, and titrated. HKC-8 cells were infected with either LV-Ctrl (1.5 × 10^8^ transduction units [TU]/mL) or LV-IGF2BP3 (3 × 10^8^ TU/mL) overnight, then incubated for 2 days as indicated. The expression of the Flag-IGF2BP3 was assessed by Western blot analysis in HKC-8 cells. In some experiments, HKC-8 cells were transfected with pDel-β-cat using the Lipofectamine 2000 reagent (Invitrogen, Thermo Fisher Scientific) as previously reported (47). The empty vector pcDNA3.1 (Invitrogen) was used as a mock transfection control. For knockdown of endogenous IGF2BP3 expression, HKC-8 cells were transfected with either control siRNA or IGF2BP3-specific siRNA. At 6 hours after transfection, cells were treated with or without Wnt3a (100 ng/mL) for another 2 days. The expression of the relevant proteins was assessed by Western blot analysis and immunofluorescence staining in HKC-8 cells. The sequences of siRNA used are described in Supplemental Table 2.

### Nuclear and cytoplasmic fractionation.

Nuclear and cytoplasmic fractionation was performed with a commercial kit (BB-3102-50T; BestBio) according to the procedures specified by the manufacturer.

### Western blot analysis.

Whole-kidney homogenates and cells were prepared with RIPA buffer containing 1% NP-40, 0.1% SDS, 100 μg/mL PMSF, and 1% Halt Protease and Phosphatase Inhibitor Single-Use Cocktail (78442; Thermo Fisher Scientific) in PBS on ice. The supernatants were collected after centrifugation at 13,000*g* at 4°C for 15 minutes, and concentration was determined with BCA protein assay (K813-5000-1; BioVision). Gel electrophoresis was performed on reduced, denatured samples. After blotting onto PVDF microporous membranes (IPVH00010; MilliporeSigma) and blocking with 5% milk, membranes were incubated with primary antibodies and HRP-conjugated secondary antibodies. The protein bands were visualized by SuperEnhanced chemiluminescence detection reagents (P1010; Applygen Technologies Inc.) and Kodak x-ray film. The primary and secondary antibodies used are listed in Supplemental Table 3. Relative protein levels of Western blots were quantified with densitometries, analyzed by ImageJ software (NIH), and reported after normalizing to the loading controls.

### qRT-PCR.

Total RNA isolation was carried out using the TRIzol RNA Isolation Reagent (Life Technologies, Thermo Fisher Scientific). The first-strand cDNA synthesis was performed by using 2 μg of RNA in 20 μL of reaction buffer using a Reverse Transcription System kit (Promega). qRT-PCR was performed on ABI PRISM 7000 Sequence Detection System (Applied Biosystems, Thermo Fisher Scientific). The PCR reaction mixture was in a 25 μL volume including 12.5 μL 2× SYBR Green PCR Master Mix (Applied Biosystems, Thermo Fisher Scientific), 5 μL diluted reverse transcription product (1:10), and 0.5 μM sense and antisense primer sets. The mRNA levels of different genes were calculated after normalization with β-actin or GAPDH. The sequences of the PCR primer pairs used are described in Supplemental Table 4.

### Renal expression of IGF2BP3 via AAV9 vector.

AAV9 vector was used as a gene transfer vehicle for overexpression of IGF2BP3 in the kidney. The recombinant AAV vector expressing IGF2BP3, designated as AAV-BP3, was constructed by Hanbio Biotechnology Co. Briefly, the full length of IGF2BP3 cDNA was inserted into a pHBAAV-CMV-MCS-3xflag-T2A-ZsGreen vector, and the accuracy of the inserted IGF2BP3 was validated by sequencing. AAV9 was packed, purified, and titrated. Male C57BL/6 mice were anesthetized and injected with AAV-Ctrl (1.9 × 10^12^ viral genomes [vg]/mL) or AAV-BP3 (1.7 × 10^12^ vg/mL) into 6 sites (10 μL at each site) of the left renal cortex with a glass micropipette (36). Pilot experiments showed that exogenous IGF2BP3 expression was highest at 12 weeks after injection. Based on this information, UUO was performed at 12 weeks after intraparenchymal microinjection of AAV. Mice were divided into 4 groups (*n* = 5 per group): (*i*) sham+AAV-Ctrl, (*ii*) UUO+AAV-Ctrl, (*iii*) UUO+AAV-BP3, and (*iv*) sham+AAV-BP3. The detailed experimental design is presented in Figure 6A. Mice were sacrificed at 7 days after UUO, and kidney tissues were collected for various analyses.

### Knockdown of IGF2BP3 expression in vivo.

Knockdown of IGF2BP3 expression in vivo was carried out by an shR-based inhibition. Briefly, IGF2BP3-specific shR expression plasmid (pLVX-IGF2BP3-shR) and empty vector (pLVX-shR) were administered into mice via tail vein injection, using an established hydrodynamics-based gene transfer approach, as described previously (7). The sequence of mouse IGF2BP3 siRNA is listed in Supplemental Table 2. Plasmid injection was carried out at 2 days after UUO. Male C57BL/6 mice were divided into 3 groups (*n* = 5 per group): (*i*) sham, (*ii*) UUO+Ctrl-shR, and (*iii*) UUO+IGF2BP3-shR. The detailed experimental design is shown in Figure 7A. Mice were sacrificed 7 days after UUO, and the kidney tissues were collected for various analyses.

### Inhibition of β-catenin signaling in vivo.

For assessing the effect of β-catenin signaling on IGF2BP3 expression in vivo, mice were divided into 3 groups (*n* = 5 per group): (*i*) sham, (*ii*) UUO injected with vehicle, and (*iii*) UUO injected with ICG-001. Mice were injected intraperitoneally with vehicle or ICG-001 at 5 mg/kg body weight once a day beginning from day 3 after UUO. Mice were sacrificed 7 days after UUO, and the kidney tissues were collected for various analyses.

### Determination of Scr levels.

Scr levels were determined by an automatic chemistry analyzer (AU480; Beckman Coulter). The levels of Scr were expressed as milligrams per deciliter.

### Histology and immunohistochemical and immunofluorescence staining.

Paraffin-embedded mouse and human kidney sections (3 μm thickness) were prepared by a routine procedure (48, 49). The sections were stained with Sirius red or Masson’s trichrome, according to the manufacturer’s protocols, respectively. Immunohistochemical staining was performed using the established protocol as described previously (50). Quantification of fibrotic lesions and positive protein area was assessed by a computer-aided point-counting technique. Briefly, about 10 random, nonoverlapping 40× cortical images were taken using an upright Olympus light microscope for each mouse and human. Integrated optical density of fibrotic lesions, positive protein area, and the whole field area were quantified using Image-Pro Plus (Media Cybernetics). The percentage of fibrotic lesions or positive protein area over the whole field area was calculated, respectively. The average value from 10 images for each mouse and human was used as the final value. HKC-8 cells cultured on coverslips were fixed with 4% paraformaldehyde for 15 minutes at room temperature, then immersed in 0.2% Triton X-100 for 10 minutes. After blocking with 10% donkey serum for 1 hour, slides were immunostained with the specified antibodies, then stained with Cy2- or Cy3-conjugated secondary antibodies. Antibodies used are summarized in Supplemental Table 3. Nuclei were stained with DAPI (C1006; Beyotime Biotechnology) for 10 minutes. Images were captured under fluorescence microscopy (Leica SP8, Leica Microsystems).

### TOPFlash luciferase reporter assay.

HEK293T cells were infected with either LV-Ctrl or LV-IGF2BP3 overnight and then incubated for 2 days as indicated. The HEK293T cells were then transfected with the TOPFlash luciferase reporter plasmid (0.9 μg, Addgene plasmid 12456) using Lipofectamine 2000 reagent. A fixed amount (0.1 μg) of internal control reporter *Renilla reniformis* luciferase driven under a thymidine kinase promoter (pRL-TK, Promega) was also cotransfected for normalizing the transfection efficiency. The luciferase assay was performed using the Dual-Luciferase Reporter Assay System kit according to the manufacturer’s protocols (E1910; Promega). Relative luciferase activity (arbitrary units) was calculated as fold-induction over controls after normalizing the transfection efficiency.

### RIP.

HKC-8 cells at 80%~90% confluence were harvested by trypsinization and subjected to RIP. Briefly, RIP was carried out by using the RIP-Assay Kit (RN1001; MBL International) according to the manufacturer’s recommendations with normal rabbit IgG/RIP-certified IGF2BP3 antibody and protein A/G plus-agarose beads (Santa Cruz Biotechnology). Western blotting of a portion of washed RIP samples was performed to confirm specificity of IP. Following RNA isolation, RIP and input RNA were quantified. The IGF2BP3 RIP usually yielded more than 500 ng of RNA while IgG control was less than 50 ng. qRT-PCR was performed as previously described with the primers of *CTNNB1* and 18S rRNA, using equal volumes of RIP eluate for IgG and IGF2BP3 for cDNA production. 18S rRNA was used for normalization.

### CTNNB1 mRNA stability.

For assessing the stability of *CTNNB1* mRNA, control and IGF2BP3-depleted HKC-8 cells were incubated with actinomycin D (HY-17559; MedChemExpress) at 5 μg/mL. Cells were then collected at different time points as indicated, and RNA was isolated for qRT-PCR. β-Actin was used for normalization.

### ISH.

*CTNNB1* probes (5′-ACTCAAGCTGATTTGATGGAGTTGGACATG-3′, 5′-GGGTTCAGATGATATAAATGTGGTCACCTG-3′, and 5′-TGCCTCCAGGTGACAGCAATCAGCTGGCCT-3′) were purchased from Boster. Paraffin-embedded kidney sections (3 μm) were fixed with 4% paraformaldehyde, and ISH was performed with a commercial kit (Boster) according to the procedures specified by the manufacturer.

### Statistics.

All data examined were expressed as mean ± SEM. Statistical analyses of the data were performed using SPSS 19.0. Comparisons between groups were made by 2-tailed *t* test or 1-way ANOVA followed by the Student-Newman-Keuls test. Spearman’s (nonparametric) correlation analysis was used to assess the relationship between IGF2BP3 area and other variables. *P* < 0.05 was considered significant.

### Study approval.

All animal studies were approved by the Animal Ethics Committee at the Nanfang Hospital, Southern Medical University. Studies involving human samples, for which written informed consent was provided for use, were approved by the Medical Ethics Committee at the Nanfang Hospital, Southern Medical University. All animal studies were in accordance with the US Public Health Service Policy on Humane Care and Use of Laboratory Animals.

## Author contributions

HF and Y Liu conceived the study. DS, HF, and Y Liu designed the experiments; DS, JS, Y Long, MZ, LL, JC, YX, HT, HZ, and XH performed the experiments; DS, HF, FFH, and Y Liu analyzed the data; FFH provided human kidney specimens; DS and Y Liu created the figures; DS and Y Liu wrote the manuscript; and all authors approved the final version of the manuscript.

## Supplementary Material

Supplemental data

## Version 1 12/15/2022

In-Press Preview

## Version 2 01/24/2023

Electronic publication
