# Supplementary material for: Insulin-like growth factor 2 mRNA-binding protein 3 promotes kidney injury by regulating β-catenin signaling
Source: JCI Insight. 2023 Jan 24;8(2):e162060. doi: 10.1172/jci.insight.162060 (PMC9977311; doi:10.1172/jci.insight.162060)
Supplement: Supplemental data [file jciinsight-8-162060-s038.pdf]

Insulin-like growth factor 2 mRNA-binding protein 3 promotes kidney injury by regulating β-catenin signaling

Dongyan Song[1,2], Jingyue Shang[1,2], Yinyi Long[1], Menghua Zhong[1], Li Li[1], Jiongcheng Chen[1], Yadie Xiang[1], Huishi Tan[1], Haili Zhu[1], Xue Hong[1], Fan Fan Hou[1], Haiyan Fu[1], Youhua Liu[1]

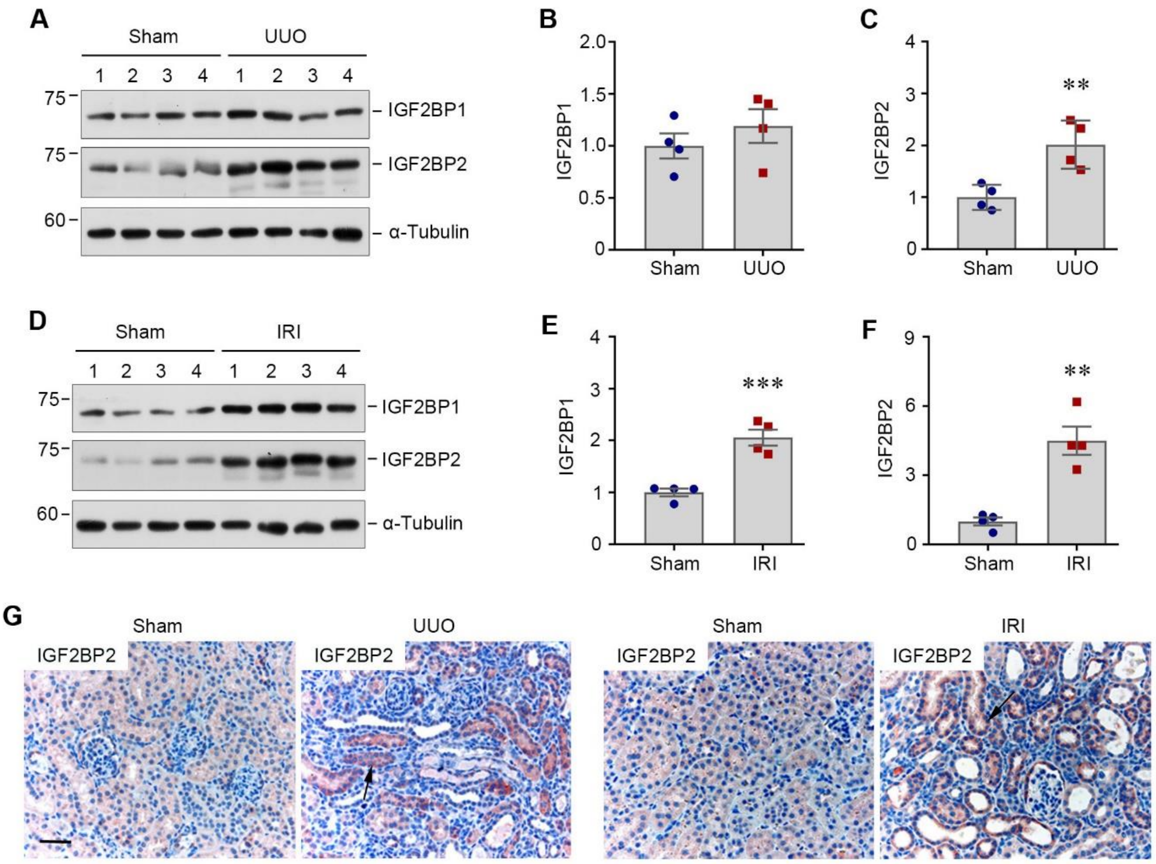

Figure S1. Renal expression of IGF2BP1 and IGF2BP2 in various models of CKD. (**A-C**) Western blot analyses (**A**) and quantitative determination of renal IGF2BP1 (**B**) and IGF2BP2 (**C**) after UUO. IGF2BP1 and IGF2BP2 expression were assessed in the kidneys at 7 days after UUO. $^{**}P < 0.01$ versus sham (n=4, *t* test). (**D-F**) Western blot analyses (**D**) and quantitative determination of renal IGF2BP1 (**E**) and IGF2BP2 (**F**) after IRI. IGF2BP1 and IGF2BP2 expression were assessed at 11 days after IRI. $^{**}P < 0.01$, $^{***}P < 0.001$ versus sham (n=4, *t* test). (**G**) Representative micrographs show renal expression and localization of IGF2BP2 protein in various animal models of CKD. IGF2BP2 was detected by immunohistochemical staining. Arrows indicated positive staining. Scale bar, 50 μm.

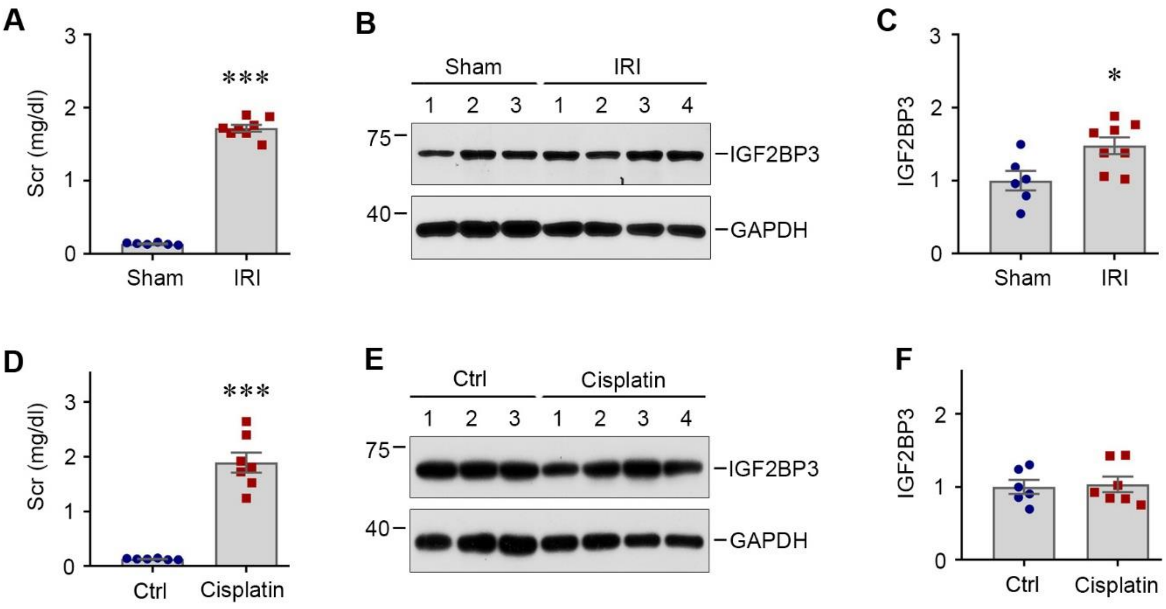

Figure S2. Renal expression of IGF2BP3 in mouse models of AKI. (**A**) Graphic presentation shows Scr levels after IRI. $^{***}P < 0.001$ versus sham (n = 6-8, *t* test). (**B** and **C**) Western blot analyses (**B**) and quantitative determination (**C**) of renal IGF2BP3 protein after IRI. IGF2BP3 expression was assessed in the kidneys at 24 h after IRI. $^{*}P < 0.05$ versus sham (n = 6-8, *t* test). (**D**) Graphic presentation shows Scr levels after cisplatin injection. $^{***}P < 0.001$ versus sham (n = 6-7, *t* test). (**E** and **F**) Western blot analyses (**E**) and quantitative determination (**F**) of renal IGF2BP3 protein after cisplatin injury. IGF2BP3 expression was assessed in the kidneys at 3 days after cisplatin (n = 6-7, *t* test).

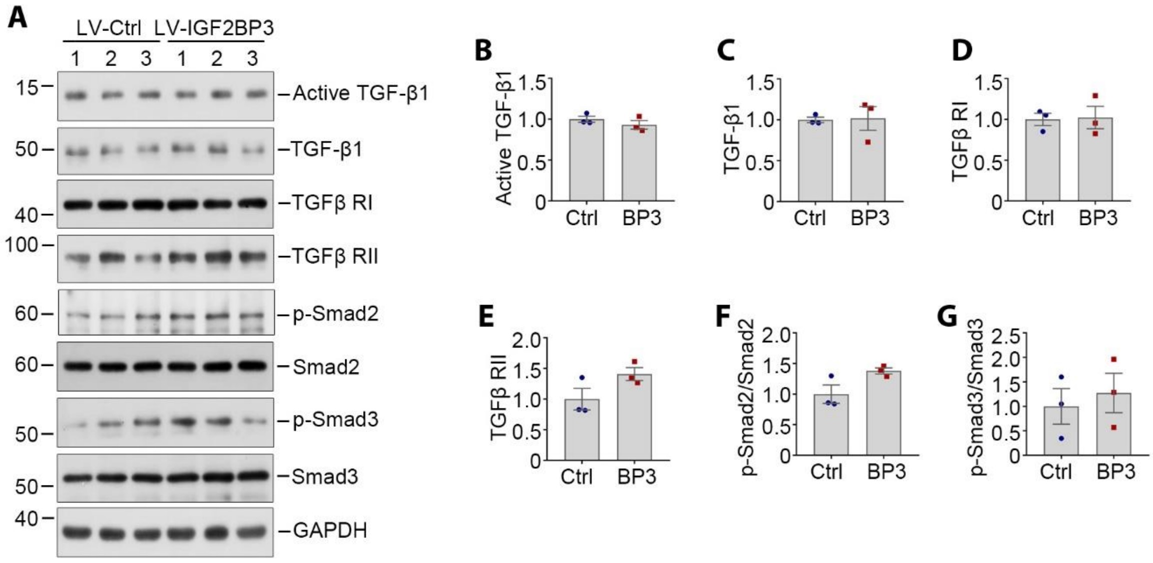

Figure S3. Overexpression of IGF2BP3 has little effect on TGF-β signaling in vitro. (**A**) HKC-8 cells were infected with either control lentivirus or IGF2BP3 lentivirus overnight, and then incubated for 2 days as indicated. Representative Western blots (**A**) and quantitative data show the expression of active TGF-β1 (**B**), TGF-β1 (**C**), TGF-β RI (**D**), TGF-β RII (**E**), p-Smad2/Smad2 (**F**), and p-Smad3/Smad3 (**G**) in different groups as indicated (n=3, *t* test).

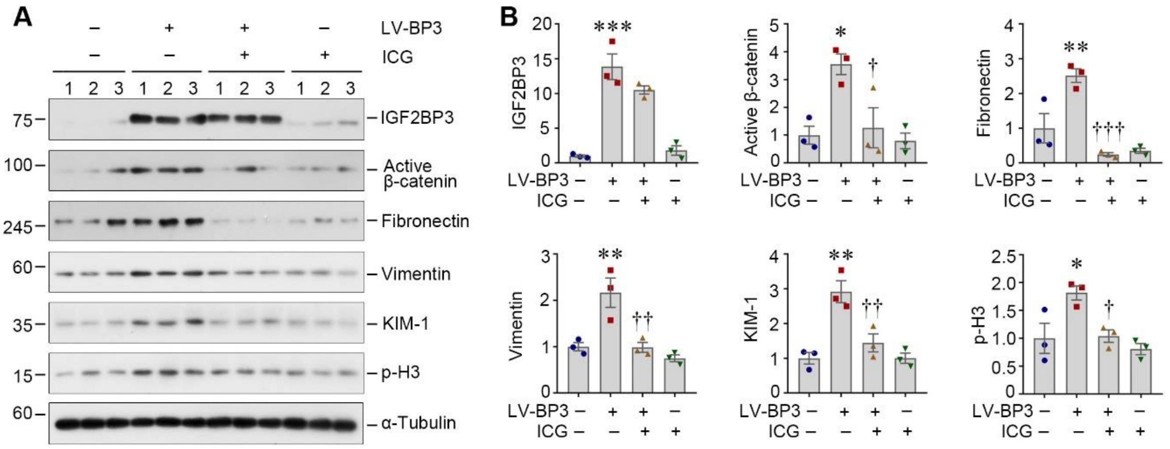

Figure S4. The effects of IGF2BP3 overexpression are mediated by β-catenin activation in vitro. HKC-8 cells were infected with either control lentivirus or IGF2BP3 lentivirus overnight, and then incubated with ICG-001 (10 μM) for 2 days as indicated. Representative Western blots (**A**) and quantitative data (**B**) show the expression of IGF2BP3, active β-catenin, fibronectin, vimentin, KIM-1, and p-H3 in different groups as indicated. $^{*}P < 0.05$, $^{**}P < 0.01$, $^{***}P < 0.001$ versus controls; $^{\dagger}P < 0.05$, $^{\dagger\dagger}P < 0.01$, $^{\dagger\dagger\dagger}P < 0.001$ versus the LV-BP3 group (n=3, Student-Newman-Kuels test).

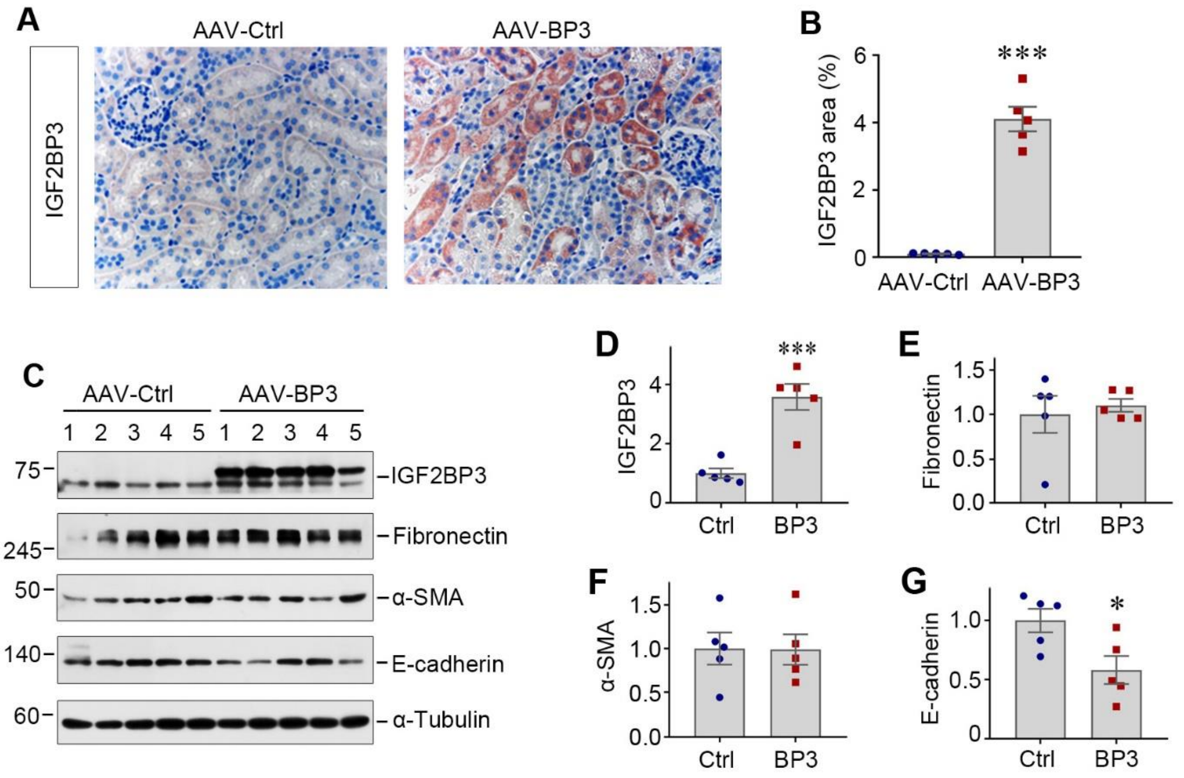

Figure S5. Mice with overexpression of IGF2BP3 are phenotypically normal under basal condition. (**A**) Representative micrographs show IGF2BP3 overexpression in renal tubular epithelium in normal mice after injection of IGF2BP3 adeno-associated virus (AAV-BP3). (**B**) Semi-quantitative determination of the expression of IGF2BP3 in the kidney of normal mice after injection of AAV-BP3. (**C**-**G**) Representative Western blot (**C**) and quantitative data show the expression of IGF2BP3 (**D**), fibronectin (**E**), α-SMA (**F**) and E-cadherin (**G**) in different groups as indicated. $^{*}P < 0.05$, $^{***}P < 0.001$ versus the sham injected with AAV-Ctrl group (n = 5, *t* test).

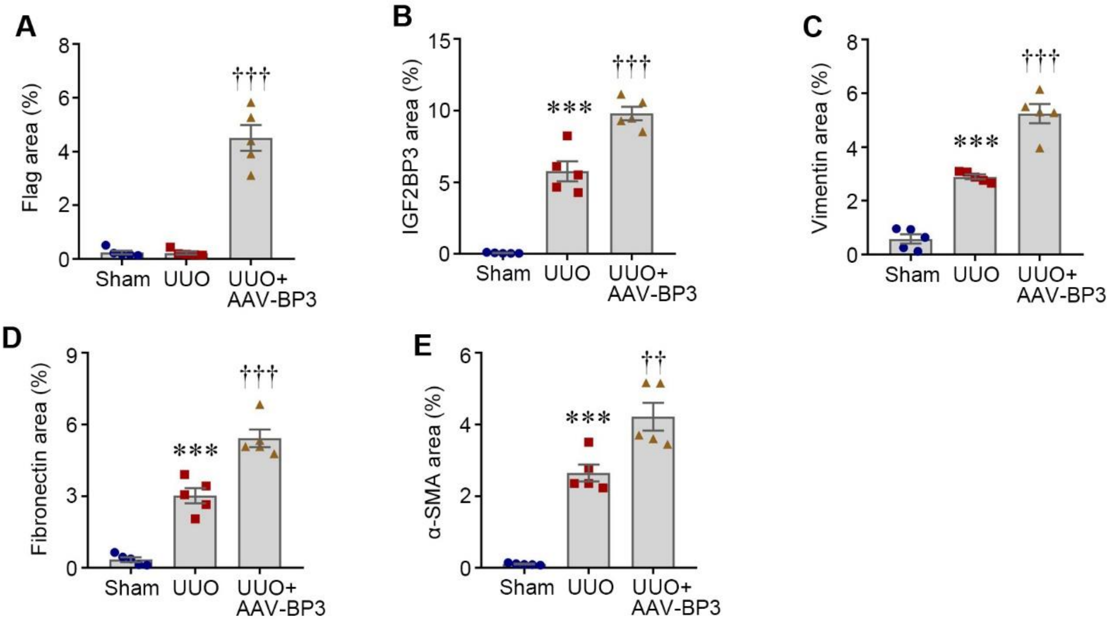

Figure S6. Overexpression of IGF2BP3 promotes renal fibrosis after UUO. (**A**-**E**) Semi-quantitative determination of Flag (**A**), IGF2BP3 (**B**), vimentin (**C**), fibronectin (**D**), and α-SMA (**E**) in different groups as indicated. $^{***}P < 0.001$ versus the sham injected with AAV-Ctrl group; $^{††}P < 0.01$, $^{†††}P < 0.001$ versus the UUO injected with AAV-Ctrl group (n = 5, Student-Newman-Kuels test).

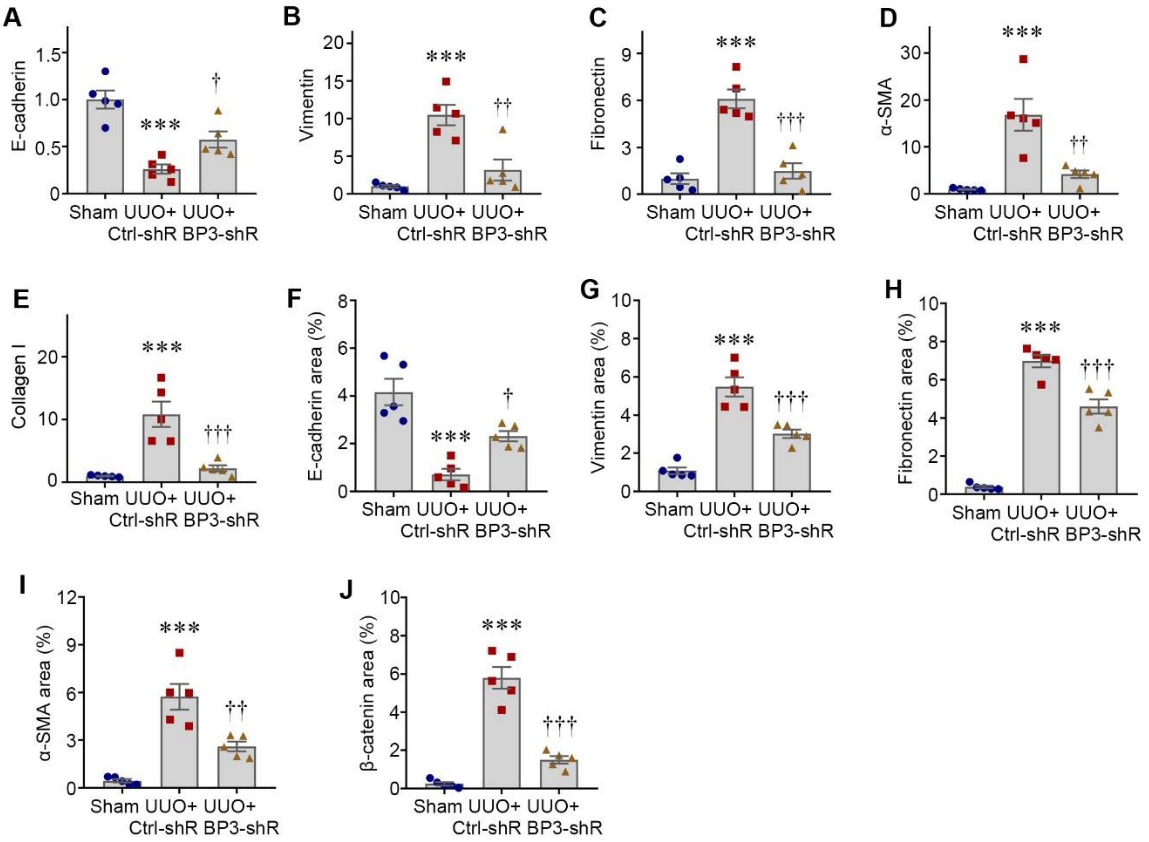

Figure S7. Knockdown of IGF2BP3 ameliorates renal fibrosis and inhibits β-catenin activation after UUO. (**A-E**) Quantitative data of Western blot analyses show the expression level of E-cadherin (**A**), vimentin (**B**), fibronectin (**C**), α-SMA (**D**) and collagen I (**E**) proteins after normalization with α-tubulin in different groups as indicated. $^{***}P < 0.001$ versus the sham group; $^{\dagger}P < 0.05$, $^{\dagger\dagger}P < 0.01$, $^{\dagger\dagger\dagger}P < 0.001$ versus the UUO injected with Ctrl-shR group (n=5, Student-Newman-Kuels test). (**F-J**) Semi-quantitative determination of E-cadherin (**F**), vimentin (**G**), fibronectin (**H**), α-SMA (**I**), and β-catenin (**J**) protein staining in different groups. $^{***}P < 0.001$ versus the sham group; $^{\dagger}P < 0.05$, $^{\dagger\dagger}P < 0.01$, $^{\dagger\dagger\dagger}P < 0.001$ versus the UUO injected with Ctrl-shR group (n=5, Student-Newman-Kuels test).

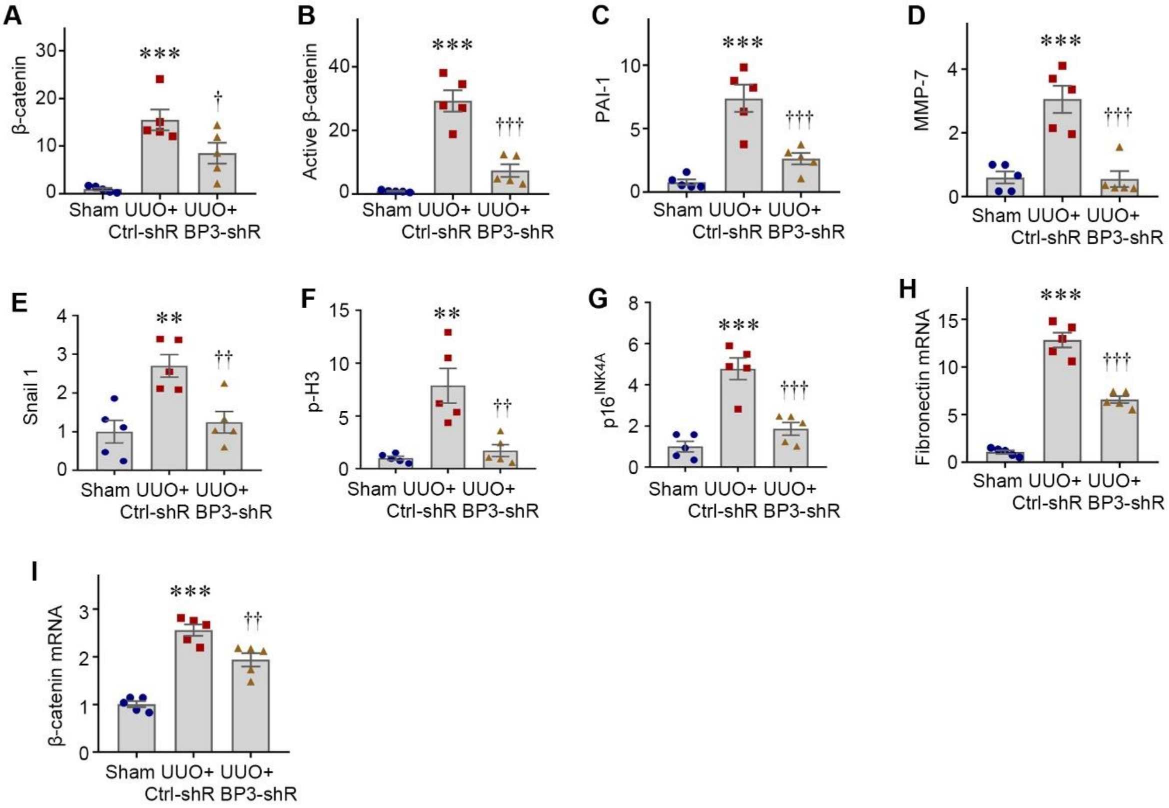

Figure S8. Knockdown of IGF2BP3 ameliorates renal fibrosis and inhibits β-catenin activation after UUO. (**A-G**) Quantitative data show the expression of β-catenin (**A**), active β-catenin (**B**), PAI-1 (**C**), MMP-7 (**D**), Snail 1 (**E**), p-H3 (**F**), and p16^{INK4A} (**G**) in different groups as indicated. $^{**}P < 0.01$, $^{***}P < 0.001$ versus the sham group; $^{\dagger}P < 0.05$, $^{\dagger\dagger}P < 0.01$, $^{\dagger\dagger\dagger}P < 0.001$ versus the UUO injected with Ctrl-shR group (n=5, Student-Newman-Kuels test). (**H** and **I**) Quantitative real-time polymerase chain reaction analyses (qRT-PCR) of renal fibronectin (**H**) and β-catenin (**I**) mRNA expression in different groups as indicated. $^{***}P < 0.001$ versus the sham group; $^{\dagger\dagger}P < 0.01$, $^{\dagger\dagger\dagger}P < 0.001$ versus the UUO injected with Ctrl-shR group (n=5, Student-Newman-Kuels test).

Table S1. Demographic and clinical data of CKD patients.

Characteristics	CKD patients
**Gender-No. (%)**	
Male	14 (56)
Female	11 (44)
**Age at entry-years**	
Mean ± SEM	43.2±17.1
Range	18-77
**Time from diagnosis to biopsy (month)**	12.0 (3.5-24.0)
**Scr (μmol/l)**	133.7±61.3
**eGFR (ml/min/1.73m^2)**	57.5±27.9
**Pathological Diagnosis-No. (%)**	
DN	4 (16)
CTIN	4 (16)
LN	4 (16)
IgAN	5 (20)
FSGS	3 (12)
MN	5 (20)

Table S2. The sequences of siRNA used to silence the indicated targets in this study.

Target	Species	Sequence	
		sense	antisense
IGF2BP3	Human	GGAUUCGGAAACUUCAGAUTT	AUCUGAAGUUUCCGAAUCCTT
Igf2bp3	Mouse	GCGGAGAAGUCCAUUACUATT	UAGUAAUGGACUUCUCCGCTT

Table S3. The sources of antibodies used in this study.

Antibodies	Catalogue number	Company	Usage	Location
**Primary antibodies**				
anti-IGF2BP3	NBP1-84339	Novus Biologicals	WB/IF (Human)	Littleton, CO
anti-IGF2BP3	Ab177477	Abcam	WB/IF (Mouse)	Cambridge, MA
anti-IGF2BP3	Ab179807	Abcam	IHC	Cambridge, MA
anti-IGF2BP3	RN009P	MBL International	RIP	Woburn, MA
anti-fibronectin	F3648	Sigma-Aldrich	WB/IHC/IF	St. Louis, MO
anti-α-SMA	A2547	Sigma-Aldrich	IHC	St. Louis, MO
anti-α-SMA	Ab5694	Abcam	WB	Cambridge, MA
anti-collagen I	BA0325	Boster Biological Technology	WB	Wuhan, China
anti-E-cadherin	Ab76055	Abcam	WB/IHC	Cambridge, MA
anti-vimentin	Ab92547	Abcam	WB/IHC	Cambridge, MA
anti-β-catenin	610154	BD Biosciences	WB/IF	San Jose, CA
anti-β-catenin	Ab15180	Abcam	IHC	Cambridge, MA
anti-active β-catenin	#19807	Cell Signaling Technology	WB	Danvers, MA
anti-MMP-7	GTX104658	GeneTex	WB	Irvine, CA
anti-PAI-1	AF3828	R & D Systems	WB	Minneapolis, MN
anti-snail 1	Ab180714	Abcam	WB	Cambridge, MA
anti-flag	F1804	Sigma-Aldrich	WB	St. Louis, MO
anti-flag	F3165	Sigma-Aldrich	IHC	St. Louis, MO
anti-IGF2BP1	22803-1-AP	Proteintech	WB	Rosemont, IL
anti-IGF2BP2	11601-1-AP	Proteintech	WB/IHC	Rosemont, IL
anti-KIM-1	BA3537	Boster Biological Technology	WB	Wuhan, China
anti-p-H3	#9701	Cell Signaling Technology	WB	Danvers, MA
anti-p16^{INK4A}	Ab189034	Abcam	WB	Cambridge, MA
anti-p53	#2524	Cell Signaling Technology	WB	Danvers, MA
anti-TGF-β1	Ab92486	Abcam	WB	Cambridge, MA
anti-TGF-β RI	Ab235178	Abcam	WB	Cambridge, MA
anti-TGF-β RII	Sc-17791	Santa Cruz Biotechnology	WB	Santa Cruz, CA
anti-p-smad2	#3104	Cell Signaling Technology	WB	Danvers, MA
anti-smad2	#5339	Cell Signaling Technology	WB	Danvers, MA
anti-p-smad3	#9520	Cell Signaling Technology	WB	Danvers, MA
anti-smad3	#9523	Cell Signaling Technology	WB	Danvers, MA
anti-α-tubulin	RM2007	Ray Antibody Biotech	WB	Peachtree Corners, GA
anti-GAPDH	RM2002	Ray Antibody Biotech	WB	Peachtree Corners, GA
anti-TBP	Ab818	Abcam	WB	Cambridge, MA
**Secondary antibodies**				
Goat anti-mouse	BA1050	Boster Biological Technology	WB	Wuhan, China
Goat anti-rabbit	BA1054	Boster Biological Technology	WB	Wuhan, China
Rabbit anti-goat	BA1060	Boster Biological Technology	WB	Wuhan, China
Donkey Anti-Mouse	715-065-150	Jackson ImmunoResearch	IHC	West Grove, PA
Donkey Anti-Rabbit	711-065-152	Jackson ImmunoResearch	IHC	West Grove, PA
Donkey Anti-Mouse	715-225-150	Jackson ImmunoResearch	IF	West Grove, PA
Donkey Anti-Rabbit	711-165-152	Jackson ImmunoResearch	IF	West Grove, PA

Table S4. The sequences of primers used for qRT-PCR in this study.

Gene	Species	Primer Sequence 5' to 3'	
		Forward	Reverse
CTNNB1	Human	GCCCTGGTGAAAATGCTTGG	CGCACTGCCATTTTAGCTCC
18S rRNA	Human	GCCCGAAGCGTTTACTTTGA	TCCATTATTCCTAGCTGCGGTATC
ACTB	Human	CTCACCATGGATGATGATATCGC	AGGAATCCTTCTGACCCATGC
Igf2bp3	Mouse	TGGAAGTTGAGCACTCGGTC	ACTTGCTCACAGCTCTCCAC
Ctnnb1	Mouse	ACTTGCCACACGTGCAATTC	ATGGTGCGTACAATGGCAGA
Fn1	Mouse	CTGGGTTGTTGGTGGGATGT	TGGGTCTTCCGACAGAATGC
Actb	Mouse	GAGCGCAAGTACTCTGTGTG	CGTTGCCAATAGTGATGACC
Gapdh	Mouse	TGACCTCAACTACATGGTCTACA	CTTCCCATTCTCGGCCTTG
